# Isolation of Extracellular Vesicles: General Methodologies and Latest Trends

**DOI:** 10.1155/2018/8545347

**Published:** 2018-01-30

**Authors:** Maria Yu. Konoshenko, Evgeniy A. Lekchnov, Alexander V. Vlassov, Pavel P. Laktionov

**Affiliations:** ^1^Institute of Chemical Biology and Fundamental Medicine, Siberian Branch, Russian Academy of Sciences, Novosibirsk 630090, Russia; ^2^Meshalkin Siberian Federal Biomedical Research Center, Ministry of Public Health of the Russian Federation, Novosibirsk 630055, Russia

## Abstract

**Background:**

Extracellular vesicles (EVs) play an essential role in the communication between cells and transport of diagnostically significant molecules. A wide diversity of approaches utilizing different biochemical properties of EVs and a lack of accepted protocols make data interpretation very challenging.

**Scope of Review:**

This review consolidates the data on the classical and state-of-the-art methods for isolation of EVs, including exosomes, highlighting the advantages and disadvantages of each method. Various characteristics of individual methods, including isolation efficiency, EV yield, properties of isolated EVs, and labor consumption are compared.

**Major Conclusions:**

A mixed population of vesicles is obtained in most studies of EVs for all used isolation methods. The properties of an analyzed sample should be taken into account when planning an experiment aimed at studying and using these vesicles. The problem of adequate EVs isolation methods still remains; it might not be possible to develop a universal EV isolation method but the available protocols can be used towards solving particular types of problems.

**General Significance:**

With the wide use of EVs for diagnosis and therapy of various diseases the evaluation of existing methods for EV isolation is one of the key problems in modern biology and medicine.

## 1. Introduction

**Figure 1:**
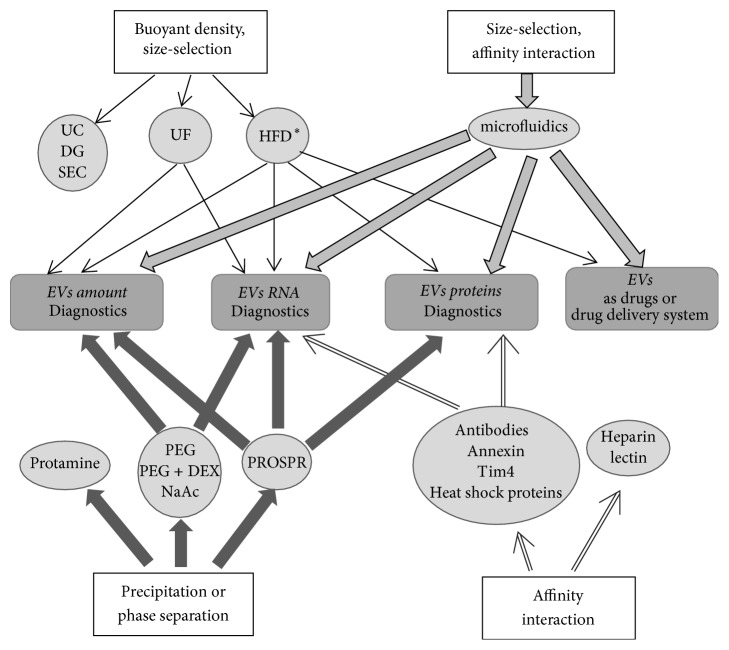
The principles used for EVs isolation, methods of isolation, and possible areas of their application. The areas of possible application depending on the properties of EVs are indicated for methods suitable for large scale isolation. ^*∗*^HFD method was designed for highly diluted samples, for example, urine. UC, ultracentrifugation, sucrose density gradient ultracentrifugation; DG, iodixanol density gradient ultracentrifugation; UF, micro- and ultrafiltration; HFD, hydrostatic dialysis; SEC, size-exclusive chromatography; PEG, EV precipitation with polyethylene glycol and commercial reagents, based on it; protamine, using EV precipitation with protamine; NaAc, using EV precipitation with NaAc; PROSPR, EV isolation via protein precipitation with organic solvent; PEG + DEX, distributive method.

Extracellular vesicles are a heterogeneous group of membrane-covered nanoparticles of diverse sizes and shapes produced by prokaryotic and eukaryotic cells. The EV membranes formed by lipid bilayer with integrated proteins protect the EV content from proteases and nucleases. As has been shown, EVs contain surface receptors, membrane and soluble proteins, lipids, ribonucleic acids (mRNA, microRNA, tRNA, rRNA, small nucleolar RNA, small circular nucleolar RNA, piRNA, scaRNA, viral RNA, Y RNA, and long noncoding RNA) [1–4], and, according to some publications, genomic and mitochondrial DNAs [4, 5]. Of special interest is the role of EVs in the communication between cells via horizontal transfer of proteins, nucleic acids, and other biologically active molecules [6]. EVs have been observed in all biological fluids of the body: blood, urine, saliva, semen, bronchoalveolar lavage, bile, ascitic fluid, breast milk, cerebrospinal fluid, and so forth [7, 8]. EVs are secreted by cells of all tissues and organs in both health and pathologies [9, 10]. Analysis of the EV contents provides the information about differentiation/functional state of parental cells. Evidently, the cells contribute with different inputs to the EVs pool of a tissue or a biological fluid. In particular, the major contributors to the pool of blood microvesicles are platelets (accounting for up to 70–90% of the blood EVs [9]). Other hematopoietic cells—namely, reticulocytes, B lymphocytes, T cells, neutrophils, mast cells, dendritic cells, and macrophages—also make their contribution as well as cells of other body tissues, for example, epithelial ones [11, 12]. The urine mainly contains EVs from various cell types forming the nephron segments, which contact the primary/secondary urine, including the EVs generated by glomerular podocytes, tubular cells [13], and other epithelial cells of the urogenital tract (bladder and prostate). When referring to EVs as the players in the communication between cells and transport of the molecules with potential diagnostic significance, researchers use a large number of terms, exosomes, ectosomes, microparticles, microvesicles, membrane particles, separated microvesicles, exosome-like particles, apoptotic vesicles, promininosomes, prostasomes, texosomes, epididimosomes, migrasomes, and oncosomes [8, 14, 15]. Moreover, these same terms describe the same vesicles, in particular, microparticles, microvesicles, membrane particles, separated microvesicles, and so forth. However, most authors distinguish exosomes, microvesicles, and apoptotic bodies as the major types [13, 16, 17]. Since most of the methods described in this review are unable to distinguish exosomes and MVs, we will use the term EVs meaning the mixture of these types of vesicles. The difficulties, when it comes to creation of the unified nomenclature, are associated in part to the current absence of standardized methods for analysis of EVs. The assessment of both EV size and concentration is technically complicated by their heterogeneity, small size (30–1000 nm), and a wide diversity of quantitative methods used for determination of these characteristics. The EV size is assessed by transmission electron microscopy (TEM) and cryoelectron microscopy and EV size and concentration together, by nanoparticle tracking analysis (NTA), tunable resistive pulse sensing (tRPS), dynamic light scattering (DLS), and high-resolution flow cytometry (hFC). It is known that the data obtained by different methods can significantly differ and even the settings of measuring devices can considerably influence the corresponding results [18]. Individual EV types differ in their size, shape, set of transported macromolecules, way of formation, and source. Exosomes are membrane-covered structures with a uniform spherical shape (according to cryoelectron microscopy data and NTA [19]) with a density in sucrose of 1.13–1.19 g/ml and a size of 30 to 150 nm [9, 10, 17] sedimented by centrifugation for at least 1 h at 100,000 ×g. Exosomes are produced during formation of multivesicular bodies and secreted into the extracellular space as a result of their fusion with the plasma membrane [9, 13]. Exosomes contain the number of specific proteins, such as tetraspanin family proteins (CD63, CD9, CD81, and CD82), flotillin, TSG101, Alix, and heat shock proteins (HSP60, HSP70, HSPA5, CCT2, and HSP90) [6, 10, 11, 17, 20]. Exosome membranes contain cholesterol, sphingomyelin, phosphatidylinositol, ceramide, lipid rafts associated to several proteins (Src tyrosine kinase, glycosylphosphatidylinositol-containing proteins, etc.), phosphatidylethanolamine, and phosphatidylserine [5, 10, 11]. Theoretically, each exosome along with the listed lipids can contain ≤100 proteins and ≤10,000 net nucleotides of nucleic acid [21]. Microvesicles (MVs) are membrane-covered vesicles of various shapes and a diameter of 50 to 1000 nm and more [16] and are formed by budding from the plasma membrane [13, 22]. The main MV protein markers are integrins, selectins, and CD40 [11]. The MV membranes contain cholesterol, diacylglycerol, and phosphatidylserine at larger amounts as compared with exosomes [19]. Apoptotic bodies differ from the other types of vesicles by a larger size (500–4000 nm) [23, 24]. They are formed during cell apoptosis [10], have heterogeneous shape and a density in sucrose of 1.16–1.28 g/ml, and are sedimented by centrifugation at 10,000–100,000 ×g. Similar to MVs, apoptotic bodies expose phosphatidylserine on their surface. The major protein markers of apoptotic bodies are histones [25], TSP, and C3b [15]. Apoptotic bodies carry fragmented genomic DNA and cell organelles, which distinguishes them from the other EV types [13, 15, 22]. As is mentioned earlier, the EV content reflects the type and functional state of parental cells and determined their different effects on the target cells depending on the composition [26]. Maturation of erythrocytes, adhesion of platelets, cell lysis, and presentation of antigens are just a few processes with EVs as mediators [27]. EVs are important players in tumor progression as carriers of carcinogenic material as well as in manifold pathologies, including neurodegenerative, autoimmune, cardiovascular, viral, and prion diseases [3, 27]. Due to this and their biochemical properties, EVs are a promising source of biomarkers for various diseases [22] and represent interest for noninvasive or minimally invasive diagnosing, assessment of antitumor therapy efficiencies, and disease prognosis. In addition, removal of circulating tumor EVs is proposed to inhibit disease progression [28]. EVs are naturally secreted by cells, stable in various body media, selectively distributed in organs and tissues, immunologically inert, and able to pass through some biological barriers due to their small size. EVs can be utilized as tissue- and organ-specific drug delivery systems (DDSs) and should be able to increase the delivery efficiency and reduce the side effects [29]. EVs are appropriate for delivering chemotherapeutics (such as doxorubicin or curcumin), therapeutic microRNAs, siRNAs, nucleic acids, and proteins [13, 30]. Note that the EVs including exosomes produced by stem cells are able to induce tissue regeneration and are applicable to treating myocardial infarction and traumas of the spinal cord, brain, and many others [3, 30]. A set of requirements to the obtained EVs should be taken into account when planning an experiment aimed to study and use these vesicles, which actually determines the method for their isolation (Figure 1). Evidently, if EVs are to be used as a source of diagnostic material, it is necessary to recover the maximal amount of vesicles, while preservation of their structure and high purity of preparations are not necessary. In this case, it is reasonable to isolate EVs by the methods that provide their maximum yield. Such EVs will be more abundant in a biological fluid that directly contacts the parental cells. For example, bronchoalveolar lavage is the most appropriate when searching for biomarkers of lung cancer and the urine, when studying the kidney, prostate, or bladder cancer [26]. In the case when EVs are planned to be used as drug delivery vehicles, it is necessary to use the methods that preserve their structure and select the source that allows for harvesting the vesicles with the specificity for the target tissue or organ. For example, the EVs intended for therapies of immune or cancer diseases are isolated from dendritic cells [31–34].

The traditional methods used for EV isolation utilize the EV properties, such as size and buoyant density, namely, ultracentrifugation [35, 36], microfiltration [37, 38], and gel filtration [39, 40]. The methods based on the fact that EVs change their solubility and/or aggregate appeared somewhat later, namely, precipitation with polyethylene glycol [41, 42], protamine [43], and sodium acetate [44]. In addition, numerous methods for isolation of EV population based on highly specific interactions with the molecules exposed on the EV surface or microfluidic technologies have recently appeared [38, 45–48]. Each of these methods has their own advantages and shortcomings, which are necessary to keep in mind when planning an experiment. We have analyzed the papers referenced in PubMed over the last several years and provide a review of the methods for EV isolation from various biological samples with highlights of their advantages and disadvantages and also compare the efficiency of new methods versus the existing conventional techniques.

## 2. Ultracentrifugation

The classical method for EV isolation utilizes the separation of particles according to their buoyant density by centrifugation. At the first stage, the particles with a high buoyant density are sedimented, such as cells, cell debris, apoptotic bodies, and aggregates of biopolymers. In order to reduce losses caused by cosedimentation and to decrease contamination of the preparations with the products of cell lysis, this step also includes several substeps, namely, centrifugation at 300–400 ×g for 10 min to sediment a main portion of the cells, at 2000 ×g to remove cell debris, and at 10,000 ×g to remove the aggregates of biopolymers, apoptotic bodies, and the other structures with the buoyant density higher than that of EVs. EVs contained in the resulting supernatant are sedimented by ultracentrifugation at >100,000 ×g (100,000–200,000 ×g) for 2 h. The non-EV proteins in the EV pellet are removed by suspending followed by repeated ultracentrifugation [35]. The obtained EV preparation is further purified and the isolated microparticles are selected according to their size by microfiltration of suspension using the filters with pore diameters of 0.1, 0.22, or 0.45 *μ*m [35, 55, 97, 98]. Note that the additional stages in EV purification (washing and microfiltration) not only increase the purity of target EVs but also decrease their quantity [39, 49]. In particular, Webber and Clayton [93] demonstrated that washing decreased the EV yield (losses caused by incomplete sedimentation and aggregation in pellet); correspondingly, the fraction thus isolated does not significantly differ in its purity (the ratio of EVs to total protein) from that without additional washing. Nonetheless, the washing of microparticles/vesicles can be necessary for isolation of the EVs intended for a certain type of downstream studies (e.g., proteomic) [35]. Note that numerous protocols for EV isolation differ not only in the number of stages but also in the conditions of differential centrifugation. There is still no unified protocol for removal of cells and cell debris when assaying different biological fluids. Low-speed centrifugation (<10,000 ×g) is occasionally used at this stage or centrifugation at 16,000 ×g. Different spinning speeds (100,000 to 200,000 ×g) are also used for final EV sedimentation [99].

The efficiency of EV isolation by centrifugation depends on many factors, such as acceleration (*g*), type of rotor and its characteristics (*k* factor, radius of rotation, and sedimentation path length), and viscosity of the sample [49–53]. Correspondingly, these parameters should be taken into account when using and adjusting the ultracentrifugation protocol in order to obtain less contaminated EV fraction and standardize the results. In particular, the EV sedimentation efficiency decreases with an increase in viscosity of the sample [50]; thus, EV isolation from the blood plasma or serum requires higher-speed ultracentrifugation and longer time as compared with a cell culture [35]. The *k* factor of a rotor should be also considered when assessing the time necessary for centrifugation of a particular sample. This factor is determined by the maximal rotation speed of a centrifuge as well as by the minimal and maximal diameters of the used rotor. The time necessary for pelleting the particles with a certain sedimentation coefficient directly depends on the *k* factor and is calculated as *t* = *k*/*s*. Two types of rotors are commonly used for EV isolation—swinging bucket (SW) and fixed angle (FA) rotors. These rotors considerably differ in their sedimentation efficiency. The SW rotor is horizontal relative to the rotation axis, whereas the FA rotor is fixed at a certain angle to the rotation axis during the overall process. Correspondingly, the sedimentation path length in an SW rotor is longer as compared with an FA rotor, and the EV sedimentation efficiency in SW rotors can be lower. It is believed that SW rotors are more appropriate for separating the particles with close sedimentation coefficients, while FA rotors are better for separation of the particles with significant difference in these coefficients. As has been experimentally demonstrated, the type of rotor influences the characteristics of isolated EV fraction, for example, the protein-to-RNA ratio [51]. Nonetheless, differential centrifugation even when SW rotors are used enables isolation of pure fractions of particles only when they considerably differ in their sedimentation rate, which depends on the buoyant density, that is, the size of EVs and the density of their content [54]. If the sedimentation rates are not sufficiently different, centrifugation produces a mixture of particles with the same buoyant density. Thus, a certain portion of small particles during successive stages of differential centrifugation is sedimented at earlier states together with larger particles, whereas a portion remains in the supernatant even after an ultracentrifugation at 200,000 ×g. For example, western blot assay involving six EV markers has shown that 40% of the vesicular proteins are present in the supernatant after ultracentrifuging urine at 200,000 ×g [99]. On the other hand, the isolated EV fraction contains a certain share of large vesicular structures and large protein aggregates [53]. Note also that an increase in the centrifugation time (to 4 h and longer) elevates the level of non-EV protein impurities in EV preparations [55]. This has a significant effect on the further analysis, especially assays of the EV proteins. Formation of EV aggregates using ultracentrifugation and compaction of the pellet at a high ultracentrifugation speed can decrease the efficiency of EV isolation. Suspension of the pellet in PBS only partially solves this problem, since not all aggregates are disintegrated, and can interfere with the EV functional integrity (destruction due to the interaction at the phase interface) [35]. It has been shown that ultracentrifugation makes it possible to isolate the EV fraction with a size of 20 to 250 nm. According to the data of different authors, the isolated EVs display the following markers: CD9, CD63, CD81, TSG101, Alix, Flotillin-1, AQP2, and FLT1. In particular, such EV fraction is appropriate for assaying RNA and microRNA.  Table 3 summarizes the data on contamination of the isolated EVs with non-EV proteins, such as albumin and uromodulin/Tamm–Horsfall protein (THP).

Within addition to the presence of contaminants in the EV preparations, important disadvantages of differential centrifugation are its long duration and the need for expensive equipment, limiting its efficacy and use in clinical studies and for diagnosis (Table 1). On the other hand, the advantages of ultracentrifugation include applicability for EV isolation from large volumes of biological fluids, it requires a relatively small set of reagents and consumables, and there are no impacts on EV except gravitational force and pipetting (as no chemicals that can potentially interfere with downstream analysis of EVs are used). Due to these advantages and good reproducibility, ultracentrifugation is the method most frequently used for EV isolation and it was utilized for studying the EVs derived from cell culture supernatants and biological fluids [4, 100–103]. A systematic analysis of the relevant literature demonstrates that 90% of the studies on EV isolation conducted before 2015 utilized ultracentrifugation [104]. However, new methods for EV isolation are becoming more frequently used. Thus, the share of the papers involving EV isolation by ultracentrifugation published during the last 3 years (2014–June 2017) decreased to 62.1% (44.9% without various modifications of the method, including density gradient or additional microfiltration; Table 2); note that ultracentrifugation was used as a control when designing new methods for EV isolation in 12% of the published papers.

### 2.1. Density Gradient Ultracentrifugation

The presence of protein aggregates, apoptotic bodies, and other nonexosomal particles in the EV fraction obtained by ultracentrifugation is the major disadvantage of this method. Density gradient ultracentrifugation is used in order to increase the efficiency of particle separation according to their buoyant density [94, 105, 106]. It is known that this method enables separation of subcellular components, such as mitochondria, peroxisomes, and endosomes [107, 108], and is regarded as one of the best methods for EV isolation [53]. Density gradient ultracentrifugation utilizes two methods for formation of the gradient, namely, a continuous density gradient (formed either during centrifugation or upfront) or a stepwise gradient (the density increases in a discrete manner), a sucrose cushion [93]. A long high-speed centrifugation results in concentration of the exosome-like vesicles in a band with close densities (exosomes, approximately 1.1–1.19 g/ml, but varying depending on the EV content); thus, EVs can be separated from proteins and nucleoproteins [54, 109, 110]. The EVs isolated by ultracentrifugation express different exosomal markers, such as CD9, CD63, CD81, TSG101, Alix, Flotillin-1, AQP2, HSP70, and FLT1 as well as some amount of non-EV proteins. As for RNA and microRNA, their purity and quantity matches the values observed in isolation by classical ultracentrifugation (see Table 3 for details). Since different EV types can have similar densities, the isolation utilizing density gradient (both stepwise and continuous) centrifugation cannot provide a pure fraction of exosomes depleted of other EV types, viruses, and some non-EV proteins (e.g., THP aggregates [39, 54, 111, 112]). As has been shown, the problem of considerable non-EV protein contamination of the target fraction can be solved by using double sucrose cushion of two layers containing 1 and 2 mol/l sucrose in D2O, respectively [109, 111]. In this case, EVs accumulate in the layer with 1 mol/l sucrose, while larger vesicles and aggregates reside in the layer with 2 mol/l. Western blot assay demonstrates that the fraction isolated using double sucrose cushion displays the protein pattern that better matches urinary exosomes as compared to the fraction isolated by ultracentrifugation or standard protocol with a sucrose cushion [109]. Standard protocols for density gradient ultracentrifugation yield the EV preparations with a higher purity as compared to a classic ultracentrifugation [35, 53, 93]. Moreover, this method gives much better results in terms of purity of EV fraction and amount of EV proteins and RNA, as compared to classical ultracentrifugation, as well as commercial kits [55]. Several studies have demonstrated that iodixanol is preferable for density gradient over sucrose, since it can form isosmotic solutions at different densities which preserve the vesicle size and shape [55] and allows for isolation of the EVs devoid of virions [113]. This method successfully separates EVs from apoptotic bodies and HIV-1 particles. The latter is an especially difficult task, since EVs and viral particles have similar densities and several surface molecules (ICAM-1, LFA-1, CD55, CD59, MHC-II, and MHC-I) [25, 114]. Axis-Shield (United Kingdom) has designed a commercial solution, OptiPrep™, 60% (wt/vol) aqueous solution of iodixanol with a density of 1.32 g/ml, intended for discontinuous density gradient. This gradient was used to isolate the EV fraction with a particle size of 50–100 nm [36]. It is also demonstrated that the EV preparations isolated using this solution lack any microvesicles over 200 nm unlike the EVs obtained by other methods [57]. Currently, density gradient ultracentrifugation is frequently used for isolation of microvesicles (Table 2). However, this method results in a considerable loss of EVs; it is complex, laborious, and time-consuming (up to 2 days) and requires expensive equipment [55, 57, 89]. Thus, this modification of ultracentrifugation technique enables production of the EV fraction of higher purity; however, similar to the classical ultracentrifugation, it is inapplicable in a clinical setting.

## 3. Filtration

The current research literature offers numerous protocols for EV isolation utilizing the separation of micro/nanoparticles according to their size [59, 115], including ultrafiltration, hydrostatic dialysis, and gel filtration.

### 3.1. Ultrafiltration

The currently available commercial membrane filters have pores of various diameters with a narrow range of pore size distribution, which simplifies isolation of the particles with a specified size. Researchers frequently supplement a method used for EV isolation with micro- or ultrafiltration. In particular, ultrafiltration may alternate successive ultracentrifugation stages [98, 103] or it can be an additional step in gel filtration chromatography [62]. However, micro- and ultrafiltration alone are also applicable for EV isolation [54, 102]. The diversity of isolation protocols used by different researchers considerably complicates comparison of the results obtained by different laboratories. When isolating EVs by microfiltration, the filters with pore diameters of 0.8, 0.45, 0.22, and 0.1 *μ*m are typically used; such filters retain the particles with diameters of over 800, 450, 220, and 100 nm, respectively (+/−20%). Larger particles are removed first (by filters with pore diameters 0.8 and 0.45 *μ*m) and the particles with a size smaller than the target EVs are separated from the filtrate at the next stage (by filters with pore diameters 0.22 and 0.1 *μ*m). Thus, the EV fraction of a specified size is concentrated. The microfiltration protocol utilizing commercially available 0.1 *μ*m hydrophilized polyvinylidene difluoride (VVLP) filter (Millipore, United States) with a low affinity for protein was proposed [116].

A protocol based on sample concentration by ultrafiltration was proposed to isolate urinary EVs [115]. The authors used ultrafiltration cells with nanomembranes that depleted the proteins with molecular weight exceeding 100 kDa, followed by centrifugation at 3000 ×g. This protocol does not use ultracentrifugation and is applicable to assay small-volume samples (e.g., 0.5 ml of urine, which is convenient volume for processing clinical archives samples). As was shown, part of microvesicles can stably bind to the membrane; thus, an additional washing of the membrane is required to reduce the EV loss [115]. The obtained fraction displays exosomal markers that are not recorded in the filtrate, thereby demonstrating retention of EVs by the nanoporous membrane. The morphology of isolated vesicles matches that of the vesicles isolated by ultracentrifugation. Later studies have shown that the EV preparations isolated by ultrafiltration display a low content of EV proteins, such as aquaporin and nephrolysin, and rather high concentration of non-EV proteins, such as albumin and *α*-1-antitrypsin [92, 117]. This can be explained by the fact that the claimed cutoffs for the molecules of certain molecular weights are typically considerably lower than the real ones; thus, this method is unable to efficiently purify the EV fraction from non-EV proteins [30], while contamination with the contaminating proteins of biological fluids considerably complicates analysis of the EV protein contents. The ultrafiltration through hydrophilized membranes with a low affinity for proteins or gel filtration is used to reduce the losses from irreversible EV binding to membranes [92, 116].

However, part of EVs stably binds to the membrane even when using the materials with a low affinity for proteins [38, 116]. Centrifugation, pressure, or vacuum is used to “push” the specimen through the membrane; however, protein molecules and newly formed aggregates of biopolymers block the membrane pores as the sample is being concentrated [38], thereby slowing the process and increasing the concentration of contaminant molecules and resulting in partial loss of the target material [51]. In addition, potential deformation of EVs caused by pressure, vacuum, and contact with membrane requires further investigation. Nonetheless, some data suggest that filtration makes it possible to isolate the EVs almost identical to those obtained by ultracentrifugation in their morphology, number, and purity with regard to non-EV proteins and presence of exosomal markers (Table 3). Note that ultrafiltration is considerably faster, simpler, and less laborious method and does not require any expensive equipment [39, 57, 115, 116]. On the other hand, Alvarez et al. [67] demonstrated that ultrafiltration, compared to ultracentrifugation and PEG precipitation of EVs, results in lower EV quantity and suboptimal RNA (including microRNA) purity.

Note that the protocols utilizing ultrafiltration in combination with centrifugation and ultracentrifugation successfully separate individual fractions of large microvesicles and exosomes in a selective manner [118]. Microfiltration through the filters with a pore diameter of 0.65 *μ*m and centrifugation at 10,000 ×g give MVs, while successive filtration using 0.65, 0.45, 0.22, and 0.1 *μ*m filters and ultracentrifugation allow for selective isolation of exosomes. The difference in the composition of isolated fractions is confirmed by cryoelectron microscopy, particle size analysis by dynamic light scattering, and western blot assays for Alix, TSG101, CD63, CD81, and EpCAM proteins. Another method for selective isolation of exosomes is the successive ultrafiltration comprising several stages, namely, filtration using 0.1 *μ*m filter (Millipore Express (PES) membrane Stericup Filter Unit with a low affinity for proteins) and five-time tangential flow filtration using 0.1 *μ*m filter (100 nm TrackEtch filter, Millipore, United States). The protocol is elaborated so that the first stage separates the exosomes and MVs from the very large particles; tangential flow filtration cleans the specimen from small-sized contaminants (mainly proteins), and the final step selectively separates exosomes and MVs [119]. The specimen was comprehensively characterized at all isolation stages using NTA in a NanoSight LM-10 instrument (Nanosight Ltd., United Kingdom) and TEM. The isolated preparation contained lower amount of EVs as compared to the specimen isolated by ultracentrifugation but had higher purity: 80% of the isolated particles corresponded to exosomes (size, <100 nm) versus 23% of analogous particles after ultracentrifugation. Proteomic analysis of the isolated EVs identified 60 different proteins, including CD63, an exosomal marker. Thus, the protocols for EV isolation from biological fluids utilizing ultrafiltration are sufficiently efficient of EVs from biological fluids. The number of filtration stages is inversely proportional to the amount of isolated EVs and directly proportional to the purity of EV preparations.

### 3.2. Hydrostatic Filtration Dialysis

Musante et al. [60] propose a protocol for MV isolation from urine samples based on hydrostatic filtration dialysis (HFD). The main advantages of this method are the lack of ultracentrifugation step and the possibility of isolating EVs from highly diluted solutions. In addition, the authors assure that this method allows avoiding EV loss. The samples are centrifuged at 2000 ×g to remove cells, bacteria, debris, and part of the THP aggregate. The supernatant is transferred to a separating funnel connected with a dialysis membrane permeable for the particles with a molecular weight of up to 1000 kDa. The fluid containing the particles of up to 1000 kDa pass through the membrane due to the hydrostatic pressure of the urine in the funnel; upon this process, undesirable components are removed from the sample and its volume decreases. Then the vesicles are sedimented by centrifugation at 40,000 ×g. The authors [60] succeeded in isolating the vesicles of 50–90 nm carrying an exosomal marker, TSG101. The EV preparations thus obtained contain mainly RNAs with a length of <1000 nucleotides and a major peak at 10–40 nucleotides; the RNA pool lacks rRNA. Later the efficiency of this method was confirmed and it was shown that an additional purification of the urine sample from bacteria was necessary to increase the accuracy of further RNA analysis [59]. Thus, hydrostatic dialysis is a more efficient and rapid method for EV isolation and provides reduced losses as compared to ultracentrifugation [61]; in addition, this method is advantageous when working with large- volume samples, such as urine. This method unifies the concentration, volume, and electrolytic composition of the sample; correspondingly, the authors propose it in processing the samples intended for storage in biobanks [61]. Note that this method in its essence is the ultrafiltration under conditions when small pressure is applied to the sample, which equals the fluid column in a dialysis cell.

### 3.3. Gel Filtration (Size Exclusion Chromatography)

Gel filtration makes it possible to separate the molecules differing in their hydrodynamic radius and is widely used for separation of biopolymers (proteins, polysaccharides, proteoglycans, etc.). As has been shown, this method is also applicable to separation of EVs from the blood plasma and urine protein complexes and lipoproteins [42, 63, 64, 120], which is challenging and many other methods fail [120]. However, a pretreatment and concentration of EV samples by ultracentrifugation [37, 92] or ultrafiltration [62, 65, 120] are necessary in order to obtain the EV preparations free of proteins and lipoprotein impurities. Western blot assay for typical microvesicular proteins aquaporin-2 and neprilysin for exosomal markers demonstrates that successive ultracentrifugation and chromatography allow for isolation of the microvesicular fraction with a relatively large representation of the markers as compared with the classical ultracentrifugation or ultrafiltration [92]. Indeed, they preserve their integrity, biological activity, and initial abundance during gel filtration, since they move with the fluid flow under a small differential pressure and, according to available data, almost do not interact with the fixed phase [38, 53]. In addition, the use of the buffers with a high ionic strength minimizes the interaction of biopolymers and nonspecific contamination of EV preparations [38]. Note also that chromatography is readily scalable: an increase in the length of columns enhances the peak resolution for the particles close in their sizes, while an increase in the column diameter allows for analysis of more concentrated samples with a larger volume (the resolution of close zones is proportional to the square root of the column length). Nonetheless, it is necessary to keep in mind that the separation efficiency depends on the volume of the analyzed sample (which should not exceed 1/20 to 1/15 of the column volume), number of components, and difference in the sizes (hydrodynamic radii) of the separated particles [38]. In order to simplify the procedure of EV isolation by gel chromatography, several types of commercial columns have been designed, in particular, qEV Size Exclusion Columns (Izon Science Ltd., United Kingdom), Sepharose 2B (Sigma, United States), Sepharose CL-4B (Sigma, United States), Sepharose CL- 2B (30 mL; GE Healthcare, Sweden), and Sephacryl S-400 (GE Healthcare, United Kingdom) [121, 122]. Comparison of EV isolation efficiency with commercial columns demonstrates that they differ in both the efficiency and the degree of contamination with albumin in the resulting EV preparations [121]. Presumably, the chemical composition and structure of the fixed phase influence the EV isolation efficiency due to the interaction of microparticles and other components of the separated mixture with the surface (nonspecific sorption) or different porosity and inner volume of the resins. The EVs isolated by gel chromatography, ultracentrifugation, and ultrafiltration have almost the same size. Western blot assay of EV markers in most studies has shown that the EVs isolated by gel chromatography display a larger number of markers as compared with ultracentrifugation, ultrafiltration, and EV precipitation with polyalcohols. The EV fraction isolated by gel chromatography displays a low content of non-EV proteins (see Table 3 for more details). Thus, gel chromatography is an efficient, rapid, and almost loss-free method with a high reproducibility enabling isolation of EVs. Since EVs and, in particular, exosomes have rather large hydrodynamic radius as compared with proteins, lipoproteins, and protein complexes, they are relatively easily separable from these components. Nonetheless, the size of some chylomicrons is close to the isolated vesicles; correspondingly, the EV preparations thus obtained contain lipoproteins but at a considerably lower rate as compared with the contamination observed in the case of other methods used for EV isolation [38, 42]. The disadvantages of this method are its low yield and rather expensive chromatographic sorbents; also, the obtained exosomal fraction is dilute and might require concentration for certain downstream applications.

## 4. Methods Utilizing the Change in EV Solubility and/or Aggregation

### 4.1. Precipitation with Hydrophilic Polymers

Analysis of the relevant publications over the last 3 years demonstrates that the method based on precipitation of EVs in PEG solutions is the second in its popularity after ultracentrifugation (26.3% of all research papers; Table 2). Indeed, PEGs with various molecular weights have been long used for precipitation of proteins, nucleic acids, viruses, and other small particles [123]. This method utilizes a decrease in the solubility of compounds in the solutions of superhydrophilic polymers, PEGs. The procedure reduces to mixing of the sample and polymer solution, incubation, and sedimentation of EVs by low-speed centrifugation (1500 ×g). The EV pellet is then suspended in PBS for further analysis. The undeniable advantages of EV precipitation with PEG are simplicity and speed as well as the possibility of working in physiological pH range and weak dependence on the ion concentration (Table 2). PEG 6000 or commercial reagents for EV isolation by PEG precipitation—ExoQuick (System Biosciences, United States), Total Exosome Isolation Reagent (Invitrogen, United States), ExoPrep (HansaBioMed, Estonia), Exosome Purification Kit (Norgen Biotek, Canada), and miRCURY Exosome Isolation Kit (Exiqon, Denmark)—can be used. The amount and quality of the EVs and microRNA isolated using self-prepared PEG 6000 optimized solution can be comparable to the commercial reagents but the cost of procedure is considerably lower [66]. The size of the EVs isolated with PEG is comparable to the particles isolated by ultracentrifugation, ultrafiltration, and gel chromatography, whereas the amount of EVs and the characteristic protein molecules and RNAs is, as a rule, significantly larger [38, 40, 57, 66]. However, EV precipitation is accompanied by coprecipitation of non-EV nucleoproteins and proteins, such as albumin, apolipoprotein E, and THP [55, 57] as well as immunoglobulins, immune complexes, and so on. On the other hand, various commercial kits isolate EVs with different efficiency and quality. For example, the mentioned Exosome RNA Isolation Kit (Norgen, Biotek Corp.) yields the fraction with a high EV content and a low concentration of contaminating proteins suitable for further analysis of both proteins and RNA [68, 105]. A modified ExoQuick protocol was proposed for isolating EVs from urine samples; unlike the standard protocol, this modification comprises centrifugation at a higher speed (17,000 versus 3000 ×g), DTT treatment of sample, heating for THP depolymerization, a large volume of ExoQuick-TC solution (3.3 versus 2 ml per 10 ml urine), and longer incubation with polymer solution [124]. The modified method gives larger amount of EVs and allows larger amount of microRNA to be isolated as compared with the standard ExoQuick-TC protocol, ultracentrifugation and ultrafiltration [124].

Unlike ultracentrifugation or gel chromatography, PEG precipitation makes it possible to concurrently process a large number of samples. The procedure is simple, fast, and scalable; does not deform EVs; and requires no additional equipment for isolation, which makes this method most attractive for clinical research. However, it also has several disadvantages that complicate further analysis. The main disadvantage of this method is variable contamination of the sample with proteins, protein complexes, lipoproteins, and nucleoproteins, as well as viral and other particles [55, 57]. In addition to large vesicles and poorly soluble protein aggregates, the EV fraction thus obtained might contain the molecules of biopolymers, which could interfere with further analysis of the sample (mass spectrometry, proteomic analysis, and RNA assay). These impurities can be removed from the sample by subsequent centrifugation, filtration, or gel filtration [38].

### 4.2. Precipitation with Protamine

All extracellular vesicles are negatively charged. This suggested use of protamine, a positively charged molecule, to aggregate and isolate EVs from the blood plasma, saliva, and cell cultures [43]. It has been shown that EVs are even more efficiently precipitated by protamine in the presence of PEG 35,000 Da. The initial stage in this protocol is centrifugation (1500–3000 ×g). Then biological samples are mixed with precipitating solutions (4 : 1), such as 1–0.1 mg/ml protamine, 0.2 g/ml PEG 35,000, or a mixture of protamine and PEG. The resulting solution is incubated overnight and centrifuged at 1500 ×g (30 min, 22°C). The pellet is suspended in buffer and gel-filtered on a Sephadex G-100 (GE Healthcare Bio-Sciences AB, Sweden) column to purify the sample from lipoproteins, other low molecular weight impurities, and protamine [125]. The amounts of EVs isolated at protamine concentrations of 1, 0.5, 0.25, and 0.1 mg/ml were compared [43]. The highest EV yield was observed for 0.25 mg/ml protamine. Addition of PEG enhances suspension of the pellet. In particular, the EV yield from different biological fluids using a mixture of protamine and PEG was higher as compared with the yields in the case of protamine or PEG alone as well as in the case of ultracentrifugation. On the other hand, the size of the EVs isolated by different methods as well as expression of exosomal markers (CD63, CD9, and CD81) was similar. Analysis of RNA and microRNA has shown that their amounts do not differ when isolated by ultracentrifugation and using the mixture of protamine and PEG and the EV biological activity is even higher for the protamine and PEG variant [43]. Thus, this method has some fundamental advantages, simplicity of the procedure, efficiency of microRNA analysis, preservation of intact EVs, and relatively low cost. As for the disadvantages, note that the procedure is rather long, suspension of the protamine precipitate is not a simple task, gel filtration is required, and the sample still might contain residual protamine.

### 4.3. EV Precipitation with Sodium Acetate

Molecules of negatively charged phosphatidylserine are exposed on the surface of EVs. A method for EV isolation by neutralizing the surface charge with sodium acetate was proposed in 2015 [44]. The authors believe that sodium acetate interferes with the hydration of EV surface, compensates the negative charge, and initiates EV aggregation via hydrophobic interactions.

This protocol includes centrifugation (500 ×g, 30 min; 12,000 ×g, 30 min) of the sample of a biological fluid (culture medium) to remove cells, debris, and large vesicles; then the supernatant is mixed with 0.1 volume of sodium acetate buffer (1.0 M pH 4.75) and incubated on ice for 30–60 min and additionally for 5 min at 37°C. EVs are sedimented by centrifugation (5000 ×g, 10 min); the pellet is washed with 0.1 M sodium acetate buffer and centrifuged under the same conditions to suspend the pellet in HBS (HEPES buffered saline). The precipitation procedure is repeated if necessary. As it was shown, EVs precipitate best at pH ~ 4.75 in 0.1 M sodium acetate [44]. The total protein in the EV fraction thus isolated was twofold higher as compared to ultracentrifugation. This is associated with nonspecific precipitation of non-EV proteins, such as *α*2-macroglobulin. Electron microscopy and western blot assay (for Alix and HSP70) showed no difference between the EV preparations isolated by sodium acetate precipitation and ultracentrifugation [44]. Thus, this method allows for EV isolation from large-volume samples, requires no expensive equipment and reagents, and does not require the final removal of used chemicals. On the other hand, contamination of the resulting EVs with non-EV proteins can hinder their further use, especially when EVs are obtained from the biological fluids, such as the blood plasma and urine.

### 4.4. Precipitation of Proteins with Organic Solvent (PROSPR)

A method for EV isolation based on precipitation of proteins with an organic solvent, PROSPR (PRotein Organic Solvent PRecipitation) rather than EV precipitation was recently proposed [73].

This method is based on protein precipitation in acetone under the conditions that retain hydrophobic vesicles in supernatant. The sample is supplemented with fourfold volume of cold acetone (−20°C) and centrifuged (3000 ×g for 1 min) and the supernatant containing EV fraction is concentrated in a vacuum concentrator [126]. Cryoelectron microscopy data demonstrates that the size and morphology of the EVs isolated using PROSPR are similar to those isolated by sucrose density gradient ultracentrifugation (particles of 20–300 nm). On the other hand, the EV fraction isolated by PROSPR technique has a higher purity and smaller amount of proteins and their aggregates. LC-MS/MS assay of proteins also demonstrates that the PROSPR technique features lower protein (in particular, albumin) contamination as compared with the fraction obtained by ultracentrifugation. In addition, the protein markers of this EV fraction match the Vesiclepedia (extracellular vesicles database) data at the level of 90.7% versus 78.0% for the proteins of the EVs isolated by sucrose density gradient ultracentrifugation. Western blot assay demonstrates that the expression level of exosomal markers (CD9, CD63, Alix, and CD81) is higher in the EVs isolated using PROSPR as compared with those obtained by ultracentrifugation [126].

Thus, PROSPR technique is efficient and simple, which is important for its wide application in clinical setting. However, this method is inconvenient for EV isolation from large volumes and requires a deeper insight. A recent study demonstrates that PROSPR technique is suboptimal compared to gel chromatography and EV precipitation with PEG 6000 in several characteristics of the resulting EV fraction. Presumably, this is associated with the EV aggregation into multivesicular structures. The authors assume that acetone interferes with the functional properties of vesicular membranes and cause their fusion [42]. Thus, the EV isolation technique based on protein precipitation using the organic solvent most likely needs further validation.

## 5. Distributive Methods

A new method for EV isolation utilizing a two-phase system with PEG and dextran is proposed aimed at solving the problem of protein contamination in the EV fraction [74]. These two polymers concurrently dissolved in aqueous solution under certain conditions form two separate phases. In this process, specific physicochemical features of the interactions between polymer molecules and EVs cause preferential accumulation of the latter in the dextran phase, while proteins and other biopolymers as well as supramolecular complexes spread between the phases with preferential accumulation in the PEG phase. Repeated extraction of biopolymers with fresh portions of PEG solution decreases the content of contaminating proteins in the EV phase: four changes of PEG decrease the protein concentration in EV phase tenfold, whereas the EV amount remains almost the same. The efficiency of EV isolation using the PEG-dextran solution is significantly higher as compared with ultracentrifugation and ExoQuick, providing the EVs with a size and morphology analogous to those obtained by ultracentrifugation and preserving the integrity of their membranes. Western blot assay for the exosomal markers CD81, CD9, and Alix as well as RT-PCR with Melan A and GAPDH has demonstrated that both the protein markers and RNA in the EVs thus isolated are at a higher concentration as compared with the EVs obtained by ultracentrifugation or ExoQuick [74, 75]. Inhibition of PCR by high concentrations of biopolymers and a high viscosity of solutions (dextran concentration should not exceed 1.5%), hindering the manipulations, are the disadvantages of this method. In general, the EV isolation by the two-phase method is promising, inexpensive, simple, and rapid and results in pure and intact EVs (Table 2).

## 6. EV Isolation Methods Utilizing Affinity Interactions

Lipids, proteins, and polysaccharides are exposed on the surface of EVs. All these substances are potential ligands for manifold molecules, including antibodies, lectins, and lipid-binding proteins. Many options of the use of the molecules specifically interacting with the molecules on the EV outer surface and thus allowing for EV isolation have been proposed.

### 6.1. Antibodies to EV Receptors

As a rule, EVs are characterized using the antibodies specifically binding receptors—tetraspanins, heat shock proteins, and MHC antigens [80, 127, 128]. Naturally, such antibodies can be used to isolate EVs; the antibodies covalently bound to the fixed phase are typically used for this purpose [129]. Magnetic beads [56, 127, 128], highly porous monolithic silica microtips [130], surface of plastic plates [129], cellulose filters [131], and membrane affinity filters [132] are also utile for this purpose. The diversity of antibodies and fixed phases has given rise to a large number of protocols for isolation of EVs. For example, Clayton et al. [127] proposed an immunomagnetic separation of B-lymphocyte exosomes from supernatants of cultivated cells. They used paramagnetic beads with a diameter of 4.5 *μ*m coated with anti-HLA DP, DQ, and DR antibodies (Dynal, Norway) incubated with conditioned culture medium for 24 h at a room temperature and isolated the EV complexes with magnetic particles with the help of a magnet. The complexes were then washed and assayed by TEM and flow cytometry using staining with the antibodies conjugated to fluorescein isothiocyanate. In the resulting preparation, exosomes with an average diameter of 70 nm accounted for 71.6% of all EVs and with a size of 100 nm and larger, for 29.4%. In its speed and simplicity the method is comparable to the traditional techniques. For example, standard ultracentrifugation followed by immunoblotting procedure requires several days to 1 week when analyzing a large number of markers and a large amount of cells for isolation of exosomes. However, analysis of the magnetic bead-exosome complexes by flow cytometry takes only 1 day and requires a relatively small number of cells (1 × 10^6^) [127].

An analogous approach utilizing magnetic beads coated with the antibodies to CD9, CD63, CD81, and EpCAM markers was used by several teams [56, 128]. In some protocols, immunoprecipitation is supplemented with initial EV isolation by precipitation with hydrophilic polymers. For example, Oksvold et al. [128] enriched the EVs from conditioned cell medium using the Total Exosome Isolation Reagent (cat. #4478359; Invitrogen, Thermo Fisher Scientific, United States). The cell medium supernatant was incubated with this reagent for 12 h at 4°C and centrifuged at 10,000 ×g for 1 h at the same temperature, and the pellet was dissolved in PBS. EVs were isolated by adding the magnetic beads of a micrometer diameter (Dynabeads, Thermo Fisher Scientific, United States) coated with the antibodies specifically towards CD9, CD63, and CD81. The EV-magnetic bead complexes were separated and washed with the help of a magnet followed by flow cytometry, TEM, or western blot analysis. The authors report that this method yields the EVs uniform in their size, morphology, and protein content and are devoid of contamination with proteins and protein aggregates. This method is well compatible with the further analysis (western blot, electron microscopy, flow cytometry, qRT-PCT, and so on).

System Biosciences proposed using magnetic particles with streptavidin and a set of biotinylated antibodies (CD9, CD63, and CD81) for EV isolation. The EVs from a biological fluid (blood plasma or serum, cell culture, urine, and liquor) are enriched by ExoQuick, ExoQuick-TC (System Biosciences, United States), or ultracentrifugation. The blood serum or concentrated EV preparations are placed into wells of a 96-well plate and incubated for at least 12 h at a room temperature. Magnetic beads are separated and washed by using a special magnetic matrix to elute the exosomes for 1 h. This approach allows for scaling of the process (Exo-Flow96 and 32 Exosome IP Kits). Another advantage of this method is a larger size of the beads (9.1 *μ*m), capable of capturing larger number of exosomes as compared with the 4.5 *μ*m beads (Dynabeads, Thermo Fisher Scientific, United States), which increases the efficiency when dealing with rare exosome subpopulations. The captured exosomes can be also eluted [129] and further assayed by electron microscopy, tracking, western blot, and so forth. Note that 50 *μ*l of the sample is sufficient for assay and that 32 or 96 samples can be concurrently analyzed, which considerably reduces the time and labor expenditures. The method based on magnetic beads is comparable in its efficiency with the traditional methods. Greening et al. [56] compared ultracentrifugation, density gradient centrifugation (OptiPrep), and immunoaffinity isolation using EpCAM (CD26) antibodies on magnetic beads. The cell medium supernatant was incubated with EpCAM magnetic beads for 4 h at 4°C; exosomes were separated and washed with PBS with using a magnet, centrifuged at 10,000 ×g for 1 h at 4°C, and eluted for further electron microscopy examination or lysed for assaying proteins by electrophoresis. The exosomes isolated by three methods had a size of 40–150 nm according to electron microscopy data and contained Alix, TSG101, and HSP70 exosomal markers according to immunoblotting. Proteomic analysis of the EVs produced by three methods demonstrates that the EVs isolated by immunoaffinity technique contain at least double amount of exosomal markers and the proteins involved in EV biogenesis and transport. Thus, the use of antibodies to EVs makes it possible to obtain highly enriched exosome preparations as compared with ultracentrifugation and density gradient ultracentrifugation. However, the authors [56] emphasize that the EV separation using density gradient has significant advantages as compared with immunoaffinity isolation associated with the availability of antibodies for analysis.

A proposed original method for EV isolation [130] utilizes highly porous monolithic silica microtips with immobilized recombinant G protein loaded with anti-CD9 antibodies (MSIA D.A.R.T.'s, Protein G tips, Thermo Fisher Scientific). The G protein has two Fc-binding domains and the used recombinant G protein differs from its natural analog by the absence of the domains for binding albumin, which minimizes nonspecific interactions. To isolate EVs, serum samples (300 *μ*l) are pipetted through a highly porous sorbent, which is three times washed by pipetting PBS, and EVs are eluted from the glass surface with urea and sodium bicarbonate solutions. The resulting EVs are appropriate for solving various problems, for example, proteomic analysis by LC/MS/MS. An automated multichannel pipette makes it possible to concurrently isolate EVs from 12 blood serum samples and the overall process takes no more than 30 min. ELISA test and LC/MS/MS demonstrated that the EV preparations obtained by immunoaffinity technique are enriched for exosomes. In addition, abundant blood proteins, such as albumin and IgG, are efficiently depleted in course of isolation [130].

Thus, this method is simple and rapid and provides pure EV preparations but is limited by the sample volume (50 *μ*l serum) and, consequently, the amount of isolated material. In addition, the expression level of CD9 marker can vary depending on the type of tissue or during a disease, requiring a set of markers to be used. The advantages of this method include reproducibility of the protocol and the possibility of its automation. The use of biotinylated anti-CD63 antibodies or biotinylated annexin 5 and the cellulose filters with covalently linked avidin molecules is described by Chen et al. [131]. The assayed serum or ocular fluid sample (5 *μ*l) was loaded on a modified filter and three times washed with PBS or annexin 5-binding buffer. Then the EVs on the cellulose filter were examined by scanning electron microscopy, ELISA, or EV lysis with MirVana RNA isolation kit (Thermo Fisher Scientific, United States) and subsequent RNA analysis. The EVs isolated with the help of anti-CD63-modified filters were larger as compared with the EVs isolated using annexin 5. In general, the RNA profiles in both methods were similar. However, the protocol with anti-CD63 antibodies yields 50% more EVs as compared to annexin 5. The EVs isolated from a small serum sample (less than 10 *μ*l) using cellulose membrane are detectable by ELISA, which is an important advantage when the amount of analyzed sample is limited.

Enderle et al. [132] compared the exosomes isolated using the commercial exoRNeasy Serum/Plasma Maxi Kit (QIAGEN, Germany), based on immunoaffinity, and the EVs isolated by ultracentrifugation. The blood plasma sample was mixed with binding buffer and loaded on a column with the membrane selectively binding exosomes. The EVs bound to filter were washed with buffer and lysed by adding QIAzol reagent. This technique takes about 1 h and allows for concentrating EVs from 4 ml of blood plasma or serum to a final volume of 14 *μ*l. A specific feature of this protocol is that the intact EVs can be eluted from the filter's surface without lyses and then concentrated either with a 100 kDa filter (Sartorius, Vivaspin) or by ultracentrifugation. According to scanning electron microscopy and NTA data, the EVs produced by this protocol and standard ultracentrifugation did not differ in their size and amount. The RNA yield in the proposed protocol varied from 1 to 10 ng/ml blood plasma, which is comparable to the yield of standard ultracentrifugation.

In general, the use of antibodies makes it possible to reduce the isolation time, elevate the purity of EV preparations, and harvest specific EV fractions [133]. Along with evident advantages of the EV isolation using antibodies, the antibodies and antibody-coated magnetic beads are expensive, isolation efficiency is insufficient, and isolation from large volumes encounters certain difficulties; this substantially limits the applicability of antibodies. In particular, antibody-coated magnetic beads are efficient in the EV isolation from cell medium but this efficiency decreases when using blood or other body fluids because of competitive inhibition of binding by other biopolymers. Note also that EVs can not be readily eluted off the complexes with antibodies, which is especially important when it is desired to obtain intact vesicles.

Another important disadvantage of the antibody-coated magnetic beads, plastic plates, columns, and other solid carriers is nonspecific sorption of nontarget EVs on the solid phase. Blocking agents can not efficiently address this problem. In addition, a high selectivity of immunoprecipitation is not always necessary [129], while a long incubation of sample with antibodies, their stability, and high price limit applicability of this method, though making it most attractive for certain projects.

### 6.2. Phosphatidylserine-Binding Proteins

Another variant of EV isolation using the agents binding to the molecules exposed on the EV surface is provided by annexin 5, a protein binding to phosphatidylserine in the presence of calcium ions. As was mentioned above, it is exposed on the surface of EVs, in particular, MVs, apoptotic bodies, and, to a less degree, exosomes [134, 135]. A method for collection of exosomes using annexin 5-coated magnetic beads (ANX-beads) was proposed [78]. The sample was incubated with ANX-beads at 4°C for 15 min in the presence of calcium ions; the exosome-magnetic bead complexes were separated using a magnetic stand and washed twice with Ca-HEPES. The annexin A5-EV complexes were detected using fluorescent staining of nucleic acids (Hoechst 3342 or pyronin Y); the presence of EV-specific nucleic acids was confirmed by RT-PCR of the B2M and CK19 transcripts. The authors did not use methods for EV characterization and did not compare the EVs with those obtained by other methods. Another type of EV isolation based on phosphatidylserine binding is the use of Tim4 protein, also able to bind phosphatidylserine on the membranes in the presence of calcium ions [135]. Tim4 is a transmembrane protein of the TIM family, involved in the regulation of the immune system, which comprises Ig-like and a Ser/Thr-rich domains. Tim4 is expressed in fibroblasts, macrophages, dendritic cells, and so forth. The Tim4 protein immobilized on the surface of magnetic beads (MagCapture Exosome Isolation Kit PS; Wako, Japan) was proposed for EV isolation from conditioned culture medium [77]. A chelating agent, ethylenediaminetetraacetic acid (EDTA), was used to dissociate the Tim4 complexes from EVs. The EV isolation efficiency of the Tim4-based method was compared with the standard ultracentrifugation and a commercial kit, Total Exosome Isolation Reagent (cat. #4478359, Invitrogen, Thermo Fisher Scientific, United States), which demonstrated that the yield in the case of the commercial kit was almost twofold higher compared to the Tim4-based variant and ultracentrifugation. Mass spectrometry of the EV preparations isolated using the Tim4-based method showed that the contamination with non-EV proteins was lower than in the case of ultracentrifugation or the commercial kit. The average EV size (106 nm) in the TIM4 affinity technique was smaller as compared to the particles obtained by ultracentrifugation or the commercial kit (136 and 183 nm, resp.). According to western blot assay, the EVs isolated using Tim4 are more enriched for CD63, CD9, and CD81 markers as compared to ultracentrifugation or Total Exosome Isolation Reagent. The qPCR assay for miR-16, miR-92a, and miR-142-3p microRNAs and GAPDH mRNA showed that the amount of exosomal RNA thus isolated was tenfold higher as compared to ultracentrifugation. ELISA demonstrated that Tim4-magnetic beads were able to bind EVs with a higher efficiency as compared to CD63-conjugated beads, suggesting that the isolation utilizing Tim4 is more efficient than that with annexin 5 (see above [131]). As for disadvantages of this method, note that the Tim4-magnetic beads should be incubated overnight at 4°C and also the materials are expensive.

### 6.3. Heparin-Modified Sorbents

An interesting approach for EV isolation proposed by Balaj et al. [79] is based on the ability of heparin to bind EVs. EVs were isolated from conditioned cell medium using an agarose sorbent with heparin, Affi-Gel® Heparin Gel (Bio-Rad), and compared with the efficiency of ultracentrifugation and ExoQuick-ТС commercial kit. The resin was incubated for at least 12 h at 4°C and the unbound agarose beads were washed off with physiological saline solution. EVs were eluted with 2 M NaCl in PBS for 12 h at 4°C and characterized using nanoparticle tracking, electron microscopy, and western blot assay (Alix). The level of protein contamination was assessed by immunoblotting with anti-BSA antibodies and the RNA concentration by qRT-PCR. The EVs isolated using the heparinized agarose were morphologically similar to the EVs obtained by a standard ultracentrifugation. NTA data demonstrated 60% recovery of the total input EVs and EV size distribution was similar to that of the EVs isolated by ultracentrifugation. The albumin concentration in the samples produced by the heparin-modified sorbent was significantly lower as compared to the samples after ultracentrifugation. As for the RNA content in the EVs isolated by three methods, it did not significantly differ. The heparinized carriers have also emerged to be suitable for isolation of exosomes from the blood plasma and other biological fluids [79]. However, the procedure is rather lengthy; in addition, the blood plasma and other biological fluids contain various heparin-binding proteins. In order to increase the EV yield when using heparinized sorbents, it is proposed to enrich the EV fractions at the initial stage of isolation by ultrafiltration through a 100 kDa filter or by gel filtration [79]. Unfortunately, this makes the isolation procedure even longer and adds complexity.

### 6.4. Binding of Heat Shock Proteins

Other EV surface antigens that can be potentially utilized for EV isolation are heat shock proteins. Ghosh et al. [80] used the peptide venceremin (Vn), specifically binding the heat shock proteins, including the proteins exposed on the surface of culture medium, blood plasma, and urine EVs. In this protocol, biotinylated Vn96 peptide (100 or 50 *μ*g/ml sample) was incubated with conditioned culture medium, blood plasma, or urine for at least 12 h at 4°C or 15 min at a room temperature. After centrifugation in a benchtop centrifuge (10,000–17,000 ×g) for 7–15 min, the EV pellet was washed several times with physiological saline to obtain the target EV preparation. The specificity and efficiency of this method were assessed by several techniques (immunoblotting, NTA, TEM, AFM, NGS of microRNA, and proteome analysis) to compare the efficiency of EV isolation with the standard sucrose density gradient ultracentrifugation and the commercial ExoQuick-TC Exosome Precipitation kit (System Biosciences, United States). The size of the EVs isolated using the Vn peptide varied from 30 to 100 nm. Western blot assay demonstrated that the content of markers (CD9, CD63, CD24, HSP70, and Alix) in the plasma and urine EVs isolated using the Vn peptide was higher as compared to the samples obtained by density gradient ultracentrifugation. The proteome and RNA (microRNA and long RNA) profiles in the EV proteins isolated by three methods did not differ. Thus, the proposed method is comparable in its efficiency to traditional approaches. Note that this method makes it possible to obtain EVs in less than 40 min and requires only standard laboratory equipment.

### 6.5. Lectins

Lectins are the proteins that reversibly, noncovalently, and highly specifically bind carbohydrate motifs of glycoproteins, proteoglycans, and glycolipids. Lectins are present in plants, animals, and microorganisms and differ in their affinity for various hydrocarbons [136]. It is known that glycosylated proteins involved in important bodily processes, for example, protein transport [137], are exposed on the EV surface. Interestingly, the glycosylation patterns can differ in the norm and pathologies [137]. A designed microarray comprising 62 different plant and fungal lectins allowed for an insight into the urine EV glycosylation profile [137]. The authors initially compared the efficiency of binding of different lectins to hydrocarbon epitopes of urine exosomes. The lectins that interact with N-acetyl glucosamine and lactosamine oligomers—wheat germ agglutinin (WGA), Lycopersicon esculentum lectin (LEL), and solanum tuberosum lectin (STL)—displayed the highest affinity; note that STL has the maximum affinity for urine EVs. To isolate EVs, urine samples were incubated with biotinylated STL (0.2 mg; Vector Laboratories) and streptavidin magnetic beads (0.1 mg; Dynabeads) for at least 12 h at 4°C. The magnetic beads were collected as described above (Section 6.1) and suspended in 50 *μ*l of buffer for further analysis. The isolation efficiency was confirmed by TEM of the magnetic beads with exosomes as well as western blot assay for the markers flotillin and CD63 and flow cytometry for the markers CD63 and AQP2. In addition, the utility of concanavalin A (ConА),* Phaseolus vulgaris* erythroagglutinin (PHA-E),* Ricinus communis* agglutinin (RCA), wheat germ agglutinin (WGA), and* Sambucus nigra* agglutinin (SNA) for isolating urine EVs was also demonstrated. Several other lectins, such as* Vicia villosa* lectin (VVL), peanut agglutinin (PNA),* Dolichos biflorus* lectin (DBL),* Maackia amurensis* agglutinin (MAA),* Phaseolus vulgaris* leucoagglutinin (PHA- L), and* Lens culinaris* agglutinin (LCA) are hardly promising for isolating urine EVs [138].

The efficiency of EV isolation from the urine of healthy donors using the STL-coated magnetic beads was examined [82] and compared with the standard techniques of ultracentrifugation and precipitation with polymers, such as ExoQuick-TC (System Biosciences, United States), Total Exosome Isolation Reagent (Thermo Fisher Scientific, United States), and Exosomal RNA Kit (Norgen Biotek, Canada). The efficiency of the compared methods was assessed according to the exosomal markers and RNA yield using Total Exosome RNA and Protein Isolation kit (Thermo Fisher Scientific, United States). RNA was assayed by BioAnalyzer (Agilent Technologies, United States). EVs were characterized by cryoelectron microscopy and western blot assay (CD9, CD10, CD63, TSG101, CD10, AIP1/Alix, AQP2, and FLT1). Microcapillary electrophoretic profiles of RNA in the samples isolated by different methods demonstrated the prevalence of small RNAs. The EV preparation isolated using Urine Exosome RNA Isolation Kit contained the maximum RNA amount (2.7 ng/ml urine) followed by ultracentrifugation and Total Exosome Isolation Reagent (0.5 ng/ml urine each). The lectin-based method and ExoQuick-TC gave the smallest yield (0.2 ng/ml urine). However, it was found that each method is capable of isolating different EV populations. For example, the lectin-based method is specific for CD9-positive EVs and least specific for AIP1- and CD26-positive EVs, whereas Exosomal RNA Kit is the best in isolation of AIP1 (Alix) positive EVs; ultracentrifugation, CD63-positive; and Total Exosome Isolation Reagent and CD10- and CD26- positive EVs. Multiplex Circulating miRNA (Abcam PLC, United Kingdom) kit was used to compare 68 microRNAs isolated from the EVs obtained by different methods and TaqMan qPCR, to finally verify them [68] (Royo et al., 2016; Cancers, 2016). The abundance of different microRNA species displayed no significant differences for individual EV isolation methods except for the lectin-based variant, which appeared to be the least efficient. Thus, the enrichment of urine EV preparations for CD9-positive exosomes is negatively correlated with the content of microRNAs [83]. The proposed method is simple, allows for isolation of EVs carrying particular markers on their surface, and is appropriate for routine immunodiagnostic procedure but fails to efficiently produce the EVs carrying RNA.

## 7. Microfluidic Devices

Microfluidic devices were designed in the second half of the last century thanks to advances in semiconductor industry. The development of microfluidic hardware commenced in the 1980s and coincided with a rapid progress in microelectronics as well as the corresponding materials and processes. The methods allowing for production of microchannels, micropumps, microvalves, and micromixers were elaborated first, and the devices found wide application in the heads of jet printers. These inventions aroused much interest in analogous areas, since microscaled processes use considerably smaller amounts of reagents and are substantially faster. Indeed, the amount of reagents can be reduced from milliliters to microliters and the time span from several hours to several seconds. In addition, microfluidic devices make it possible to obtain some results unachievable by other methods. In particular, massive DNA sequencing is impossible without microfluidic hardware. Microfluidic devices are compact units composed of a network of microchannels with different diameters of tens to hundreds of micrometers capable of handling viscous media within a concentration range of pico- to microliters. Depending on the particular function, microchannels can be connected with each other. Additional specialized units can be used for fine-tuning of fluid movement. Microfluidic devices have a tremendous potential and are able to reproduce numerous laboratory processes on a microscale with a high accuracy and specificity (lab-on-a-chip) replacing expensive equipment [83]. The microfluidic devices based on immunoaffinity principle and utilizing the antibodies to EV receptors [46, 84, 86, 96] as well as the devices with microporous filtration system [85], acoustic nanofiltration [71, 139], and porous micropillars [140] have been designed for EV isolation from cell culture and various biological fluids. Many researchers tend to use an integrated approach, for example, a combination of EVs isolation and their subsequent analysis [45]. Note that the devices utilizing affinity-based isolation have higher specificity and yield purer preparations of EV subpopulations [133]. Microfluidic devices have several advantages as compared to other methods, since they allow for a considerable decrease in the necessary amount of sample, reagents, and time required for experiment as well as for automation of the process [83, 141]. However, some problems are yet to be resolved; for example, the analyzed sample can block channels. The sample input and EV yield in immunoaffinity devices or microporous filtration systems are considerably lower as compared to the traditional isolation methods [133], thereby decreasing their diagnostic potential.

## 8. Specific Features of EV Isolation from Urine

The urine EVs are a convenient source of material for diagnosing genitourinary diseases [4]. In addition, the amount and concentration of non-EV proteins in the urine in the norm is 0.033 mg/ml, which is considerably lower compared to blood plasma. The major urinary proteins include serum albumin, THP (uromodulin), aquaporin-1, aquaporin-2, uroplakin, and apolipoproteins (see [60] for review). As is known, THP, a glycoprotein with a molecular weight of 85 kDa, is the most abundant urinary protein [142, 143] with a daily excretion of 50–100 mg/day [144]. THP forms a 3D polymeric network at an acid pH in the presence of calcium and potassium ions [145]. The gel-like structure formed in the body by THP catches bacteria and interferes with the spread of infection [146]. In addition, THP is capable of binding calcium ions [147]. The THP forms complexes with negatively charged membranes via the bridges of calcium ions and the formed gel-like network retains EVs; this eventually decreases the EV yield [94]. Several methods have been proposed to release the EVs from their complex with THP, such as treatment with dithiothreitol (DTT) or CHAPS, a detergent, and salting-out [61, 109, 138, 148–150].

The most widespread method is DTT treatment, which reduces the THP ZP module, responsible for polymerization of filaments [149]. THP depolymerization in the presence of DTT results in release of the vesicles connected with the polymeric network [37, 39, 91, 95, 106, 148, 151, 152]; though widely used, DTT treatment has an essential shortcoming; namely, the THP monomers and part of polymers remain in the isolated EV fraction and interfere with the further analysis of EV components. In addition, DTT is capable of reducing disulfide bonds in proteins altering the native structure of proteins and their complexes on the EV surface, which can also influence the results of EV proteomic analysis. Moreover, DTT is not always effective [109].

CHAPS, 3-((3-cholamidopropyl)-dimethylammonio)-1-propanesulfonate, is a zwitterionic detergent destructing protein-protein interactions and dissolving THP complexes without affecting the EV functional and morphological integrity. DTT treatment of the urine successfully eliminates the main part of THP and albumin from EV preparations [37, 148]. It is known that the salting-out with 0.58 M NaCl precipitates urinal mucoproteins, including THP. This method is rapid and simple and decreases the THP content in EV fraction without altering the EV properties [138]. It has been also proposed to destroy THP aggregates by changing the urine pH to alkaline values and decreasing the divalent ion concentrations with EDTA [88]. After removing cells from the urine by centrifugation, the supernatant was diluted with cold 20 mM Tris-HCl (pH 8.6) or 20 mM Tris-HCl (pH 8.6) + 20 mM EDTA (pH 9.0) and incubated at 4°C for 1.5 min. The samples were centrifuged and the supernatant was filtered through 1.2 *μ*m filters to sediment EVs by ultracentrifugation. The resulting EVs were characterized by TEM, NTA, and western blot assay.

The RNA from EVs was isolated using a miRNEasy Micro Kit (QIAGEN, Germany) and assayed by a 2100 BioAnalyzer, (Agilent Technologies, United States) and Qubit fluorometer (ThermoFischer Scientific). The experimental and control samples were similar in their size and morphology; in addition, the RNA contents in the EV preparations in both cases were similar too. However, the EV samples isolated using the above described method contained twofold–sevenfold larger amount of EV proteins according to western blot assay.

Thus, several currently proposed methods for releasing EVs from the complex with THP are still insufficiently effective for solving this problem.

## 9. Conclusions

Since vesicles of various types and microparticles not covered by membrane display common properties, a mixed population of vesicles/nanoparticles is obtained in most studies of EVs independently of the used isolation methods [22]. Note that standardization of the protocols used in individual laboratories is essential in case of any isolation method. A wide diversity of the protocols even within the same approach for EV isolation interferes with verification, comparison, and analysis of the data obtained by different research teams. It is critical to comprehensively study the protocols for EV isolation as well as to standardize the characterization of the obtained EV preparations. It is stated that a combination of several methods (TEM, NTA, dynamic light scattering, flow cytometry, and immunohistochemical analysis for the markers specific of an isolated EV type) should be used to characterize the EV morphology, biochemical composition, and the receptors expressed by the vesicles [19]. In addition, it is required to take into account the properties of an analyzed sample when using a particular method for isolation of EVs, since the protocol should be fit to specific characteristics of the sample, such as viscosity (when analyzing the blood plasma and serum), presence of specific proteins (e.g., THP in the urine), EV concentration, and the type of further analysis/use of the isolated EVs. It is known that different methods can result in different EV subpopulations. Moreover, the EV isolation efficiency by different methods depends on the nature of biological fluids (Table 3).

Different methods have certain advantages and disadvantages (Table 2). An ideal method for isolation of EVs should be relatively simple and inexpensive and should not require a complex or expensive equipment and should be relatively fast and allow for isolation of EVs from a large number of samples. Overall, it might not be possible to develop a universal method for EV isolation but the available standard methods applicable towards solving particular types of problems should be developed and approved. Development of such methods for EV isolation aimed at applying them in diverse scientific and clinical studies is currently a high priority task.

## Figures and Tables

**Table 1:** Main advantages and disadvantages of the currently available methods for EV isolation.

Method	Time	Advantages	Disadvantages	References
*(1.1) Ultracentrifugation*, differential centrifugation: 300 ×g, 10000 ×g, 100000–200000 ×g (1.5 h)	140–600 min	Cost (in the case of ultracentrifugation), isolation from large volumes, absence of additional chemicals	Equipment, complexity, nonexosomal impurities, low reproducibility, low RNA yield, damage of exosomes; efficiency is affected by the type of rotor, force *g*, sample viscosity; only six samples can be concurrently processed in one ultracentrifuge	[35, 49–54]

*(1.2) Density gradient ultracentrifugation*, sucrose or iodixanol density gradient, differential centrifugation	250 min–2 days	Pure preparations; no contamination with viral particles after iodixanol centrifugation; absence of additional chemicals	Complexity, loss of sample, ultracentrifugation; fails to separate large vesicles with similar sedimentation rates; contamination with viral particles after sucrose density gradient procedure	[36–39, 55, 56]

*(2.1) Ultrafiltration*, nanomembrane or filters with a pore diameter of 0.8–0.1 *µ*m	130 min	Simple procedure allowing for concurrent processing of many samples; pure preparations; additional chemicals; no limitations on sample volume	Filter plugging, loss of sample, contamination (proteins); deformation of vesicles; small quantity of exosomal proteins	[37, 38, 57, 58]

*(2.2) Hydrostatic dialysis*, membrane separation at concentration gradient	30 min 1 h per 75 ml	Appropriate for analysis of highly diluted samples (urine); cost; no additional chemicals; standardizes sample concentration, volume, electrolyte composition	Need in additional urine sample purification from bacteria	[59–61]

*(2.3) Size-exclusive chromatography (SEC)*, columns filled with polymers with heterogeneous pores	1 ml/min + column washing	Reproducibility and purity; preserves vesicle integrity; use of the buffers with a high ionic strength enhances elimination of nonspecific impurities; high sensitivity, no losses, scalability, large amount of exosomal proteins; prevents EV aggregation; insensitive to high viscosity of samples; no additional chemicals	Limitations on sample volume and number of separated peaks (necessary difference of the components in molecular weight, ≥10%); specialized equipment; complexity; coisolation of large protein aggregates and lipoproteins; processing no more than one sample in each procedure; cost	[37–40, 42, 53, 57, 62–65]

*(3.1.1) Precipitation with polymers*, polyethylene glycol caused EV precipitation	65 min	Cost and simplicity of procedure; preservation of EV integrity; no need in additional equipment; pH close to physiological range; high ion concentrations	Contamination and retention of the polymer	[42, 66]

*(3.1.2) Commercial kits for polymer precipitation (ExoQuick, TEI, and Norgen)*, polymer precipitates EVs	45–65 min (sometimes overnight)	Simple procedure; preservation of EV integrity; no need in additional equipment; pH close to physiological range; high ion concentrations	Cost (especially for diluted samples, such as urine); poor reproducibility; impurities and retention of polymer; low content of exosomal proteins	[37, 39, 40, 55, 57, 66–72]

*(3.2) Precipitation with protamine*	55 min + incubation (overnight)	Cost; simple procedure; preservation of EV integrity and biological activity; purity; efficiency	Need in purification of the isolated fraction from protamine and lipoproteins (heparin + gel filtration); long duration	[43]

*(3.3) Precipitation with sodium acetate*, pH ~ 4.75, 0.1 M acetate	130 min	Cost; simple procedure; and the possibility of processing samples of large volume	Contamination with non-EV proteins	[44]

*(3.4) Precipitation of proteins with organic solvent PROSPR*, cold acetone	105 min	Cost and simplicity	Aggregation in multivesicles	[42, 73]

*(4) Two-phase isolation*, incubation in PEG-dextran mixture	75–195 min	Cost; simple procedure; no EV deformation; purity; efficiency	Repeated replacement of PEG phase and presence of polymer	[74, 75]

*(5.1) Use of antibodies to EV receptors*, in particular, tetraspanins (CD9, CD63, CD81), TSG101, EpCAM	about 240 min	Purity and high selectivity	High selectivity, cost, availability of antibodies; difficulties with detachment of molecules and analysis of intact vesicles (eluting buffers can damage EV functional activity); nonspecific binding	[36–38, 40, 47, 56, 69, 76]

*(5.2) Use of phosphatidylserine-binding proteins* (annexin and Tim4)	12 h incubation	Readily reversible binding and simplicity	Cost	[77, 78]

*(5.3) Use of heparin-modified sorbents*	24 h incubation	Cost and preservation of EV functional integrity	Need in the initial purification and concentration (ultracentrifugation)	[79]

*(5.4) Binding of heat shock proteins*	<1 h	Preservation of EV functional integrity	Cost	[80, 81]

*(5.5) Use of lectins*	12 h incubation	Cost, simplicity, purity	Need in the initial purification and concentration (ultracentrifugation of centrifugation at 20000 ×g)	[82]

*(6) Microfluidic technologies*	1–14 *µ*l/min	Rapidness, purity, efficiency	Complexity of devices and need in additional equipment; cost	[45, 46, 48, 76, 83–87]

*(7) KeepEX*, protocol for urine dilution	<2 h	Higher EV yield as compared with ultracentrifugation	Equipment, laborious procedure, limitation on the number of concurrently processed samples (to six sample)	[88]

**Table 2:** Distribution of original research papers on EV isolation.

Method	Number of papers	Rate (%) of papers
(1.1) Ultracentrifugation with modifications	172	66.6
(1.2) Ultracentrifugation	118	45.7
(1.3) Density gradient ultracentrifugation	30	11.6
(2.1) Ultrafiltration	14	5.4
(2.2) Hydrostatic dialysis	2	0.7
(2.3) Size-exclusive chromatography	8	3.1
(3.1) Precipitation with polymers (PEG)	68	26.4
(3.2) Precipitation with protamine	1	0.4
(3.3) Precipitation with acetate	1	0.4
(3.4) Precipitation of proteins with organic solvent PROSPR	3	1.2
(4) Two-phase isolation	2	0.8
(5.1) Immunoprecipitation	5	1.9
(5.2) Annexin A5 coated magnetic beads	1	0.4
(5.3) Column-based affinity method	1	0.4
(5.4) Paper-based immunoaffinity devices	1	0.4
(5.5) Lectin binding	3	1.2
(5.6) Heparin binding	1	0.4
(5.7) Tim4 affinity-based method	1	0.4
(5.8) Vn96 binding	2	0.8
(6) Microfluidic devices	9	3.5

**Table 3:** Characterization and comparison of different methods for EV isolation according to EV size and amount, representation of EV markers, and data on the proteins, RNA, and microRNA contained in isolated EV preparations.

	EV size	EV quantity	EV markers	Protein	RNA	miRNA
*(1.1) Ultracentrifugation (UС)*	20–100 nm; on the average, 53 nm (cell medium) [51] 40–100 nm + larger EVs (cell medium) [36] 50–250 nm (cell medium) [57] <300 nm; mainly, 30–150 nm (cell medium and plasma) [89] 80.9 ± 10.9 and 90.1 ± 10.0 nm (cell medium) [90] 70–110 nm (plasma and sedum) [90] Peak at 53.42 ± 1.60 nm (urine) [91]	UC < EXQ (cell medium) [90] UC < UF (cell medium) [57] UC < TEI (cell medium and plasma) [89] UC < UF, SEC < EXQ = TEI = PEG (plasma) [66] SEC < SEC + DG = UC < EXQ (urine) [39] UC < HFD (urine) [60] EXQ, UCsuc < UF = UC < EXQmodif (urine) [67]	HSP70 and flotillin-1: UC = UF TSG101: UC < UF (cell medium) [57] CD9: UC = EXQ (cell medium) [90] CD9, CD63 CD81, TSG101 (cell culture) [51] CD81, CD63, CD9, TSG101, annexin 5 (cell culture and plasma) [89] CD63: EXQ = TEI = PEG = SEC =UF = UC; no CD81 and Tsg101 (plasma) [66] Flotillin, CD63 (plasma and serum) [90]) Alix, flotillin-1, CD9: UC = EXQ < SEC = SEC + DG (urine) [39] TSG101: UC < HFD (urine) [60] AQP2 and neprilysin: UF (none) < UC < UC + SEC (urine) [92] High levels of CD63, CD9, AQP2; medium, of CD10 and FLT1; and low, of AIP1 (urine) [68] Alix and TSG101: EXQ < UF < UC = UCsucr = EXQmodif (urine) [67]	Purity (ratio of EV to protein amounts): UC < UCsucr (cell culture) [93] Amount: UC < EXQ (cell medium) [90] Amount: UC, UCsucr < EXQmodif, EXQ, UF purity: UF < EXQ < EXQmodif < UC, UCsucr (urine) [67] SEC < SEC + ODG < DUC = EXQ (urine) [39] Albumin: UC < UF THP: UC = UF (urine) [70] Albumin: UC + SEC < UF, UC (urine) [92] THP: TEI < UC lectin < Norgen (urine) [68] Proteins: UC < EpCAM < SEC < EXQ (ascites) [40]	Peaks at 160–189 and 300–400 nt (cell culture) [51] Amount: UC < TEI, mainly 200 nt and certain share of longer ones, including 18S and 28S rRNA (cell couture and plasma) [89] Amount of sRNA: UC < Norgen; high rate of tRNA (plasma); sRNA: NOR = UC (serum) [90] Lectin < UC, TEI < Norgen (2.7 ng/ml urine) ACTB1, SOD1, GNB2L1, GAPDH, EEF1A1, RPL6 transcripts: UC, TEI, lectin < Norgen (urine) [68] Amount and purity: EXQ = UF < UC = UCsucr < EXQmodif (urine) [67] Purity: UC < SEC = EpCAM < EXQ Amount: UC, EpCAM = SEC < EXQ (ascites) [40]	Amount: UC < TEI (cell culture and plasma) [89] Amount of miRNA: Norgen = UC (plasma and serum) [90] Amount and purity UF < EXQ = UC = UCsucr < EXQmodif (urine) [67]

*(1.2) Sucrose density gradient ultracentrifugation (UCsucr)*	104 ± 9.85 (cell medium) [93] 20–100 nm (urine) [94]	UCsucr < PROSPR (plasma) [73] EXQ, UCsucr < UF = UC < EXQmodif (urine) [67]	CD9, CD63, Alix, CD81: UCsucr < PROSPR (plasma) [73] Alix and TSG101: EXQ (none) < UF < UC = UCsucr = EXQmodif (urine) [67] Alix, CD9, TSG101, HSP70, AQP2 (urine) [94]	Purity (EV to protein amounts): UC < UCsucr (cell culture) [93] Albumin and other blood plasma proteins: PROSPR < UCsucr (plasma) [73] Amount: UC, UCsucr < EXQmodif, EXQ, UF Purity: UF < EXQ < EXQmodif < UC, UCsucr (urine) [67] THP (urine) [58]	Amount and purity: EXQ = UF < UC = UCsucr < EXOmodif (urine) [67]	Amount and purity: UF < EXQ = UC = UCsucr < EXQmodif (urine) [67]

*(1.3) Iodixanol density gradient ultracentrifugation (DG)*	50–100 nm (cell medium) [36] EXQ = UF = SEC + particles > 200 nm Unlike DG, where they are absent (cell medium) [57]	UC, DG < EpCAM (cell medium) [36] DG < SEC < EXQ, UF (cell medium) [57]	TSG101, FAM125B, Rab11B, tetraspanin 8, proteins TFRC, PTGFRN: UC < DG Alix, TSG101, HSP70: UC, DG < EpCAM (cell culture) [36] HSP70, flotillin-1, TSG101: EXQ < UF < SEC, DG (cell medium) [57]	Purity (ratio per 1 *μ*g protein): UF, EXQ (coisolation of non-EV protein) < DG (loss of sample) < SEC (cell medium) [57]		

*(2.1) Ultrafiltration (UF)*	50–250 nm (cell medium) [57] Peak of 70.57 ± 4.73 nm (urine) [91] UC = UF (urine) peak of 115 ± 3 nm [58]	UC < UF (cell medium) [57] UC < UF, SEC < EXQ = TEI = PEG (plasma) [66] SEC < UF, EXQ (plasma) [57] EXQ, UCsuc < UF = UC < EXQmodif (urine) [67]	HSP70 and flotillin-1: UC = UF TSG101: UC < UF (cell medium) [57] Flotillin-1: EXQ, UF < SEC (plasma) [57] CD63: EXQ = TEI = PEG = SEC = UF = UC (plasma) [66] CD24 and AQP2: UF < UC (urine) [58] AQP2 and neprilysin: UF (none) < UC < UC + SEC (urine) [92] EXQ < UF < UC = UCsucr = EXQmodif (urine) [67]	Albumin: SEC < UF, EXQ (plasma) [57] Amount: UC, UCsucr < EXQmodif EXQ, UF Purity: UF < EXQ < EXQmodif < UC, UCsucr (urine) [67] Albumin: UC < UF THP: UC = UF (urine) [58] Albumin: UC + SEC < UF, UC (urine) [92]	Amount and purity: EXQ = UF < UC = UCsucr < EXOmodif (urine) [67]	Amount and purity: EXQ = UF < UC = UCsucr < EXOmodif (urine) [67]

*(2.2) Hydrostatic dialysis (HFD)* (urine) [60]	50–90 nm	UC < HFD	TSG101: UC < HFD	Most soluble proteins and THP are absent	Enrichment < 1000 nt; rRNA is absent	miRNA (10–40 nt) is present in sRNA (6–150 nt) fraction

*(2.3) Size-exclusive chromatography (SEC)*	EXQ = UF = SEC + particles of >200 nm unlike DG, where they are absent (cell medium) [57] 80–200 nm (plasma) [42] 70–500 nm (platelet-free plasma) [63] SEC = EXQ = UF= 50–200 nm, mainly 50–100 nm (plasma) [57] On the average, 246 ± 69 nm (urine) and 250 ± 28 nm (concentrated urine) [64]	DG < SEC < EXQ, UF (cell medium) [57] UC < UF, SEC < EXQ = TEI = PEG (plasma) [66] SEC < PEG, PROSPR (plasma) [42] UC < SEC (ascites) [40] SEC < SEC + DG = UC < EXQ (urine) [39] SEC < UF, EXQ (plasma) [57]	HSP70, flotillin-1, TSG101: EXQ < UF < SEC, DG (cell medium) [57] CD63 and CD9 (plasma) [63] Flotillin-1: EXQ, UF < SEC (plasma) [57] Flotillin-1 is present and calnexin is not (plasma) [57] CD63: EXQ = TEI = PEG = SEC = UF = UC (plasma) [66] CD63 and CD9 (urine) [64] Alix, flotillin-1, CD9: UC = EXQ < SEC = SEC + DG (urine) [39] AQP2 and neprilysin: UF (none) < UC < UC + SEC (urine) [92]	Purity (ratio per 1 *μ*g protein): UF, EXQ (coisolation of non-EV protein) < DG (loss of sample) < SEC (cell medium) [57] Albumin: SEC < UF, EXQ (plasma) [57] Amount: SEC < PROSPR < PEG (plasma) [42] SEC < SEC + DG < DG = EXQ (urine) [39] Albumin: UC + SEC < UF, UC (urine) [92] THP and albumin are absent (urine) [64] Protein amount: UC < EpCAM < SEC < EXQ (ascites) [40]	Purity: UC < SEC = EpCAM < EXQ Amount: UC, EpCAM = SEC < EXQ (ascites) [40]	

*(3.1) Polyethylene glycol (PEG)*	Average size: EXQ = TEI = PEG (plasma) [66] 80–200 nm; average size, 140 nm (plasma) [42]	UC < UF, SEC < EXQ = TEI = PEG (plasma) [66] SEC < PEG, PROSPR (plasma) [42]	CD81 and Tsg101 (plasma); CD63: EXQ = TEI = PEG = SEC = UF = UC (plasma) [66] LGALS3BP, CD5L, CD9 (plasma) [42]	Amount: SEC < PROSPR < PEG (plasma) [42]		Expression of eight miRNAs: TEI = EXQ = PEG (plasma) [66]

*(3.2) TEI*	Average size: EXQ = TEI = PEG (plasma) [66] Mainly 30–150 nm, <300 nm (cell culture and plasma) [89]	UC < TEI (cell culture and plasma) [89] UC < UF, SEC < EXQ = TEI = PEG (plasma) [66]	CD81, CD63, CD9, TSG101, annexin 5 (cell culture and plasma) [89] CD81 and TSG101; CD63: EXQ = TEI = PEG = SEC = UF = UC (plasma) [66] CD9, CD10, CD26, CD63, TSG101, CD10, AIP1/Alix, AQP2, FLT1: high level of CD26 and CD63; low level of AQP2 and FLT1 (urine) [68]	THP: TEI < UC, lectin < Norgen (urine) [68]	Amount: UC < TEI, mainly 200 nt and certain share of longer ones, including 18S and 28S rRNA (cell culture and plasma) [89] Lectin < UC, TEI < Norgen (2.7 ng/ml urine) ACTB1, SOD1, GNB2L1, GAPDH, EEF1A1, RPL6 transcripts: UC, TEI, lectin < Norgen (urine) [68]	Expression of eight miRNAs: TEI = EXQ = PEG (plasma) [66] Amount: UC < TEI (cell culture and plasma) [89] miR-21 and miR-375: Norgen < TEI (urine) [95]

*(3.3) ExoQuick (EXQ)*	92.8 ± 13.8 and 78.0 ± 7.1 nm (cell medium) [90] EXQ = UF = SEC + particles of >200 nm Unlike DG, where they are absent (cell medium) [57] SEC = EXQ = UF = 50–200 nm; mainly, 50–100 nm (plasma) [57] Average size: EXQ = TEI = PEG (plasma) [66] Peak of 83.80 ± 3.67 nm (urine) [91]	UC < EXQ (cell medium) [90] DG < SEC < EXQ, UF (cell medium) [57] SEC < UF, EXQ (plasma) [57] UC < UF, SEC < EXQ = TEI = PEG (plasma) [66] EXQ, UCsucr < UF = UC < EXQmodif (urine) [67] SEC < SEC + DG = UC < EQ (urine) [39]	CD9: UC = EXQ (cell medium) [90] HSP70, flotillin-1, TSG101: EXQ < UF < SEC, DG (cell medium) [57] Flotillin-1: EXQ, UF < SEC (plasma) [57] Flotillin-1 and calnexin are absent (plasma) [57] CD81 and TSG101; CD63: EXQ = TEI = PEG = SEC = UF = UC (plasma) [66] Alix, flotillin-1, CD9: UC = EXQ < SEC = SEC + DG (urine) [39] Alix and TSG101: EXQ < UF < UC = UCsucr = EXQmodif (urine) [67]	Amount: UC < EXQ (cell medium) [90] Purity (ratio per 1 *μ*g protein): UF, EXQ (coisolation of non-EV protein) < DG (loss of sample) < SEC (cell medium) [57] Albumin: SEC < UF, EXQ (plasma) [57] Amount: UC, UCsucr < EXQmodif, EXQ, UF purity: UF < EXQ < EXQmodif < UC, UCsucr (urine) [67] SEC < SEC + DG < UC = EXQ (urine) [39] Amount of proteins: UC < EpCAM < SEC < EXQ (ascites) [40]	Amount and purity: EXQ = UF < UC = UCsucr < EXQmodif (urine) [67] Purity: UC < SEC = EpCAM < EXQ Amount: UC, EpCAM = SEC < EXQ (ascites) [40]	Expression of eight miRNAs: TEI = EXQ = PEG (plasma) [66] Amount and purity: UF < EXQ = UC = UCsucr < EXQmodif (urine) [67] miRNA amount: EpCAM < EXQ (ascites) [40]

*(3.4) Norgen*			High levels of AIP1 and CD26; medium, of CD10, FLT1, CD9, TSG101; and low, of AQP2 and CD63 (urine) [68]	THP: TEI < UC, lectin < Norgen (urine) [68]	Amount of sRNA: UC < Norgen; high rate of tRNA (plasma) [90] sRNA: NOR = UC (serum) [90] Lectin < UC, TEI < Norgen (2.7 ng/ml urine) ACTB1, SOD1, GNB2L1, GAPDH, EEF1A1, RPL6 transcripts: UC, TEI, lectin < Norgen (urine) [68]	Amount of miRNA: Norgen = UC (plasma and serum) [90] miR-21 and miR-375: Norgen < TEI (urine) [95]

*(3.5) Precipitation with protamine*		Protamine = UC (cell culture) [43]	CD63, CD9, CD81: protamine = UC (cell culture) [43]		Amount: protamine = UC (cell culture) [43]	Amount: protamine = UC (cell culture) [43]

*(3.6) Precipitation with sodium acetate (NaAc)*	NaAc = UC (cell culture) [44]		Alix and HSP70: NaAc = UC (cell culture) [44]	Amount: UC < NaAc, contamination with no-EV proteins (cell culture) [44]		

*(3.7) Precipitation of proteins with organic solvent (PROSPR)*	PROSPR = UCsucr = 20–300 nm + aggregates of vesicles of different sizes + nonmembrane particles of 30 nm 30 nm (plasma) [73] 74% particles ≤ 100 nm; 8.5% particles < 300 nm; 3.5%, < 500 nm; and 13%, >500 nm (CNS) [73]	UCsucr < PROSPR (plasma) [73] SEC < PEG, PROSPR (plasma) [42]	CD9, CD63, Alix, CD81: UCsucr < PROSPR (plasma) [73] CD9, CD63, CD81, LGALS3BP, CD5L are undetectable (plasma) [42]	Amount: SEC < PROSPR < PEG (plasma) [42] Albumin and other blood plasma proteins: PROSPR < UCsucr (plasma) [73] Amount of vesicular proteins is 7.2-fold larger and of exosomal proteins, 6.9-fold larger as compared with UCsucr (CNS) [73]		

*(4) Two-phase isolation (PEG + DEX)*	PEG + DEX = UC (mixture of exosomes and proteins) [74, 75]	UC < PEG + DEX (mixture of exosomes and proteins) [74] UC < EXQ < PEG + DEX (mixture of exosomes and proteins) [75]	CD81: UC < PEG + DEX (mixture of exosomes and proteins) [74] CD81, CD9, Alix: UC < EXQ < PEG + DEX (mixture of exosomes and proteins, mouse plasma) [75]		Amount: Melan A and GAPDH, UC < EXQ < PEG + DEX (mixture of exosomes and proteins, mouse plasma) [74, 75]	

*(5.1) Antibodies*	40–150 nm (cell medium) [56]	Anti-EpCAM (CG336) antibodies ob magnetic beads > UC, DG (cell medium) [56] UC, DG < EpCAM (cell medium) [36]	Alix, TSG101, HSP70 (cell medium) [56]	Anti-EpCAM (CG336) antibodies ob magnetic beads > DG > UC (cell medium) [56] Amount of proteins: UC < EpCAM < SEC < EXQ (ascites) [40]	Purity: UC < SEC = EpCAM < EXQ Amount: UC, EpCAM = SEC < EXQ (ascites) [40]	miRNA amount: EpCAM < EXQ (ascites) [40]

*(5.2) Annexin* (cell medium and blood plasma) [78]			EV fluorescent staining (Hoechst 3342 or pyronin Y)		Detection of B2M and CK19 transcripts (RT-PCR)	

*(5.3) Lectins*		Lectin = UC (urine) [82]	High level of CD9; medium level of TSG101, AIP1/Alix, FLT1; low level of AIP1, CD10, CD26, AQP2, CD63 (urine) [82]	THP: TEI < UC, lectin < Norgen (urine) [82] THP: TEI < UC, lectin < Norgen (urine) [68]	Lectin < UC, TEI < Norgen (2.7 ng/ml urine) ACTB1, SOD1, GNB2L1, GAPDH, EEF1A1, RPL6 transcripts: UC, TEI, lectin < Norgen (urine) [82] Lectin < UC, TEI < Norgen (2.7 ng/ml urine) ACTB1, SOD1, GNB2L1, GAPDH, EEF1A1, RPL6 transcripts: UC, TEI, lectin < Norgen (urine) [68]	

*(5.4) Heparin* (cell medium and blood plasma) [79]	Heparin sorbent = UC		Heparin sorbent = UC, ExoQuick-ТС	Heparin sorbent < UC	Heparin sorbent = UC, ExoQuick-ТС (GAPDH, EGFR, LINE1, RPL11, CD63, cMyc)	

*(5.5) Tim protein* (cell medium) [77]	106 nm	Total Exosome Isolation Reagent > TIM4	TIM4 > Total Exosome Isolation Reagent, UC (CD63, CD9, CD81)	Total Exosome Isolation Reagent, UC > TIM4	TIM4 > UC (GAPDH)	TIM4 > UC (miR-16, miR-92a, miR-142-3p)

*(5.6) Vn peptide* (cell medium, blood plasma, urine) [80]	30–100 nm	Vn peptide = UCsucr	Vn peptide > UСsucr (CD9, CD63, CD24, HSP70, Alix)		Vn peptide = UСsucr, ExoQuick-TC	Vn peptide = UСsucr, ExoQuick-TCU

*(6) Microfluidic devices*	75 ± 15 nm (cell culture and blood plasma) [84] 100 nm (blood serum and glioblastoma multiforme) [96] 150 nm (whole blood) [85]		CD63 and EpCAM (cell culture and blood plasma) [84] CD63 (blood serum and glioblastoma multiforme) [96] CD9, CD63, CD81 (whole blood) [85]		GAPDH and IDH-1 (blood serum and glioblastoma multiforme) [96] Purity (RNA/protein ratio): UC > microfluidic filtration system RNA amount (Melan A): UC = microfluidic filtration system (whole blood) [85]	

*(7) KeepEX* (urine) [88]	KeepEX = UC	KeepEX > UC	KeepEX > UC (CD9, PDX, CD59, CD63)		KeepEX = UC	

UC, ultracentrifugation; UCsucr, sucrose density gradient ultracentrifugation; DG, iodixanol density gradient ultracentrifugation; UF, micro- and ultrafiltration; HFD, hydrostatic dialysis; SEC, size-exclusive chromatography; EV, precipitation with hydrophilic polymers; PEG, with polyethylene glycol; EXQ, using ExoQuick (System Bioscience, United States); TEI, using Total Exosome Isolation Kit (Invitrogen/ThermoFisher Scientific, United States); Norgen, Exosome RNA Isolation Kit (Norgen, Biotek Corp.); protamine, using EV precipitation with protamine; NaAc, using EV precipitation with NaAc; PROSPR, EV isolation via protein precipitation with organic solvent; PEG + DEX, distributive method; and EpCAM, isolation using antibodies to EpCAM.

## References

[1] Camussi G., Deregibus M. C., Bruno S., Grange C., Fonsato V., Tetta C. (2011). Exosome/microvesicle-mediated epigenetic reprogramming of cells. *American Journal of Cancer Research*.

[2] Abels E. R., Breakefield X. O. (2016). Introduction to Extracellular Vesicles: Biogenesis, RNA Cargo Selection, Content, Release, and Uptake. *Cellular and Molecular Neurobiology*.

[3] Iraci N., Leonardi T., Gessler F., Vega B., Pluchino S. (2016). Focus on extracellular vesicles: Physiological role and signalling properties of extracellular membrane vesicles. *International Journal of Molecular Sciences*.

[4] Bryzgunova O. E., Zaripov M. M., Skvortsova T. E. (2016). Comparative study of extracellular vesicles from the urine of healthy individuals and prostate cancer patients. *PLoS ONE*.

[5] Tamkovich S. N., Tutanov O. S., Laktionov P. P. (2016). Exosomes: Generation, structure, transport, biological activity, and diagnostic application. *Biologicheskie membrany*.

[6] Borges F. T., Melo S. A., Özdemir B. C. (2013). TGF-beta1-containing exosomes from injured epithelial cells activate fibroblasts to initiate tissue regenerative responses and fibrosis. *Journal of the American Society of Nephrology*.

[7] Hu G., Drescher K. M., Chen X. M. (2012). Exosomal miRNAs: biological properties and therapeutic potential. *Frontiers in Genetics*.

[8] Gonda D. D., Akers J. C., Kim R. (2013). Neuro-oncologic applications of exosomes, microvesicles, and other nano-sized extracellular particles. *Neurosurgery*.

[9] Żmigrodzka M., Guzera M., Miśkiewicz A., Jagielski D., Winnicka A. (2016). The biology of extracellular vesicles with focus on platelet microparticles and their role in cancer development and progression. *Tumor Biology*.

[10] Ciardiello C., Cavallini L., Spinelli C. (2016). Focus on extracellular vesicles: New frontiers of cell-to-cell communication in cancer. *International Journal of Molecular Sciences*.

[11] Théry C., Ostrowski M., Segura E. (2009). Membrane vesicles as conveyors of immune responses. *Nature Reviews Immunology*.

[12] Frydrychowicz M., Kolecka-Bednarczyk A., Madejczyk M., Yasar S., Dworacki G. (2015). Exosomes-structure, biogenesis and biological role in non-small-cell lung cancer. *Scandinavian Journal of Immunology*.

[13] Mathivanan S., Ji H., Simpson R. J. (2010). Exosomes: extracellular organelles important in intercellular communication. *Journal of Proteomics*.

[14] Al-Nedawi K., Meehan B., Rak J. (2009). Microvesicles: messengers and mediators of tumor progression. *Cell Cycle*.

[15] Akers J. C., Gonda D., Kim R., Carter B. S., Chen C. C. (2013). Biogenesis of extracellular vesicles (EV): exosomes, microvesicles, retrovirus-like vesicles, and apoptotic bodies. *Journal of Neuro-Oncology*.

[16] Willms E., Johansson H. J., Mäger I. (2016). Cells release subpopulations of exosomes with distinct molecular and biological properties. *Scientific Reports*.

[17] Ha D., Yang N., Nadithe V. (2016). Exosomes as therapeutic drug carriers and delivery vehicles across biological membranes: current perspectives and future challenges. *Acta Pharmaceutica Sinica B (APSB)*.

[18] Maas S. L. N., De Vrij J., Van Der Vlist E. J. (2015). Possibilities and limitations of current technologies for quantification of biological extracellular vesicles and synthetic mimics. *Journal of Controlled Release*.

[19] Colombo M., Raposo G., Théry C. (2014). Biogenesis, secretion, and intercellular interactions of exosomes and other extracellular vesicles. *Annual Review of Cell and Developmental Biology*.

[20] Yokoi A., Yoshioka Y., Ochiya T. (2015). Towards the realization of clinical extracellular vesicle diagnostics: Challenges and opportunities. *Expert Review of Molecular Diagnostics*.

[21] Vlassov A. V., Magdaleno S., Setterquist R., Conrad R. (2012). Exosomes: current knowledge of their composition, biological functions, and diagnostic and therapeutic potentials. *Biochimica et Biophysica Acta*.

[22] Boukouris S., Mathivanan S. (2015). Exosomes in bodily fluids are a highly stable resource of disease biomarkers. *Proteomics - Clinical Applications*.

[23] Ihara T., Yamamoto T., Sugamata M., Okumura H., Ueno Y. (1998). The process of ultrastructural changes from nuclei to apoptotic body. *Virchows Archiv*.

[24] Hristov M., Erl W., Linder S., Weber P. C. (2004). Apoptotic bodies from endothelial cells enhance the number and initiate the differentiation of human endothelial progenitor cells in vitro. *Blood*.

[25] Théry C., Boussac M., Véron P. (2001). Proteomic analysis of dendritic cell-derived exosomes: a secreted subcellular compartment distinct from apoptotic vesicles. *The Journal of Immunology*.

[26] Yáñez-Mó M., Siljander P. R., Andreu Z. (2015). Biological properties of extracellular vesicles and their physiological functions. J Extracell Vesicles. *Journal of Extracellular Vesicles*.

[27] Kaur A., Leishangthem G. D., Bhat P., Mahajan V., Dev Singh N., Banga H. S. (2014). Role of Exosomes in Pathology. *Journal of Environmental Pathology, Toxicology and Oncology*.

[28] Marleau A. M., Chen C.-S., Joyce J. A., Tullis R. H. (2012). Exosome removal as a therapeutic adjuvant in cancer. *Journal of Translational Medicine*.

[29] Kim S. M., Kim H. S. (2017). Engineering of extracellular vesicles as drug delivery vehicles. Stem Cell Investig. 2017;4:74. *Stem Cell Investig*.

[30] Junker K., Heinzelmann J., Beckham C., Ochiya T., Jenster G. (2016). Extracellular Vesicles and Their Role in Urologic Malignancies. *European Urology*.

[31] Escudier B., Dorval T., Chaput N. (2005). Vaccination of metastatic melanoma patients with autologous dendritic cell (DC) derived-exosomes: results of the first phase 1 clinical trial. *Journal of Translational Medicine*.

[32] Lo Cicero A., Stahl P. D., Raposo G. (2015). Extracellular vesicles shuffling intercellular messages: for good or for bad. *Current Opinion in Cell Biology*.

[33] Pitt J. M., Charrier M., Viaud S. (2014). Dendritic cell-derived exosomes as immunotherapies in the fight against cancer. *The Journal of Immunology*.

[34] Viaud S., Terme M., Flament C. (2009). Dendritic cell-derived exosomes promote natural killer cell activation and proliferation: a role for NKG2D ligands and IL-15R*α*. *PLoS ONE*.

[35] Thery C., Amigorena S., Raposo G., Clayton A. (2006). solation and characterization of exosomes from cell culture supernatants and biological fluids. *Current Protocols in Cell Biology*.

[36] Tauro B. J., Greening D. W., Mathias R. A. (2012). Comparison of ultracentrifugation, density gradient separation, and immunoaffinity capture methods for isolating human colon cancer cell line LIM1863-derived exosomes. *Methods*.

[37] Salih M., Zietse R., Hoorn E. J. (2014). Urinary extracellular vesicles and the kidney: biomarkers and beyond. *American Journal of Physiology-Renal Physiology*.

[38] Taylor D. D., Shah S. (2015). Methods of isolating extracellular vesicles impact down-stream analyses of their cargoes. *Methods*.

[39] Daveloose D. (2016). *Isolation and characterization of urinary exosomes in metastatic castration- refractory prostate cancer [Master, thesis]*.

[40] Taylor D. D., Zacharias W., Gercel-Taylor C. (2011). Exosome isolation for proteomic analyses and RNA profiling. *Methods in Molecular Biology*.

[41] Colombet J., Robin A., Lavie L., Bettarel Y., Cauchie H. M., Sime-Ngando T. (2007). Virioplankton 'pegylation': Use of PEG (polyethylene glycol) to concentrate and purify viruses in pelagic ecosystems. *Journal of Microbiological Methods*.

[42] Gámez-Valero A., Monguió-Tortajada M., Carreras-Planella L., Franquesa M., Beyer K., Borràs F. E. (2016). Size-Exclusion Chromatography-based isolation minimally alters Extracellular Vesicles' characteristics compared to precipitating agents. *Scientific Reports*.

[43] Deregibus M. C., Figliolini F., D'Antico S. (2016). Charge-based precipitation of extracellular vesicles. *International Journal of Molecular Medicine*.

[44] Brownlee Z., Lynn K. D., Thorpe P. E., Schroit A. J. (2014). A novel "salting-out" procedure for the isolation of tumor-derived exosomes. *Journal of Immunological Methods*.

[45] He M., Crow J., Roth M., Zeng Y., Godwin A. K. (2014). Integrated immunoisolation and protein analysis of circulating exosomes using microfluidic technology. *Lab on a Chip *.

[46] Kanwar S. S., Dunlay C. J., Simeone D. M., Nagrath S. (2014). Microfluidic device (ExoChip) for on-chip isolation, quantification and characterization of circulating exosomes. *Lab on a Chip*.

[47] Kreimer S., Belov A. M., Ghiran I., Murthy S. K., Frank D. A., Ivanov A. R. (2015). Mass-spectrometry-based molecular characterization of extracellular vesicles: Lipidomics and proteomics. *Journal of Proteome Research*.

[48] Santana S. M., Antonyak M. A., Cerione R. A., Kirby B. J. (2014). Microfluidic isolation of cancer-cell-derived microvesicles from hetergeneous extracellular shed vesicle populations. *Biomedical Microdevices*.

[49] Livshits M. A., Khomyakova E., Evtushenko E. G. (2015). Isolation of exosomes by differential centrifugation: Theoretical analysis of a commonly used protocol. *Scientific Reports*.

[50] Momen-Heravi F., Balaj L., Alian S. (2013). Current methods for the isolation of extracellular vesicles. *biological chemistry*.

[51] Cvjetkovic A., Lötvall J., Lässer C. (2014). The influence of rotor type and centrifugation time on the yield and purity of extracellular vesicles. *Journal of Extracellular Vesicles*.

[52] Jeppesen D. K., Hvam M. L., Primdahl-Bengtson B. (2014). Comparative analysis of discrete exosome fractions obtained by differential centrifugation. *Journal of Extracellular Vesicles*.

[53] Abramowicz A., Widlak P., Pietrowska M. (2016). Proteomic analysis of exosomal cargo: The challenge of high purity vesicle isolation. *Molecular BioSystems*.

[54] Witwer K. W., Buzás E. I., Bemis L. T. (2013). Standardization of sample collection, isolation and analysis methods in extracellular vesicle research. *Journal of Extracellular Vesicles*.

[55] Van Deun J., Mestdagh P., Sormunen R. (2014). The impact of disparate isolation methods for extracellular vesicles on downstream RNA profiling. *Journal of Extracellular Vesicles (JEV)*.

[56] Greening D. W., Xu R., Ji H., Tauro B. J., Simpson R. J. (2015). A protocol for exosome isolation and characterization: Evaluation of ultracentrifugation, density-gradient separation, and immunoaffinity capture methods. *Methods in Molecular Biology*.

[57] Lobb R. J., Becker M., Wen Wen S. (2015). Optimized exosome isolation protocol for cell culture supernatant and human plasma. *Journal of Extracellular Vesicles*.

[58] Gerlach J. Q., Krüger A., Gallogly S. (2013). Surface Glycosylation Profiles of Urine Extracellular Vesicles. *PLoS ONE*.

[59] Tataruch-Weinert D., Musante L., Kretz O., Holthofer H. (2016). Urinary extracellular vesicles for RNA extraction: optimization of a protocol devoid of prokaryote contamination. *Journal of Extracellular Vesicles (JEV)*.

[60] Musante L., Tataruch D. E., Holthofer H. (2014). Use and isolation of urinary exosomes as biomarkers for diabetic nephropathy. *Frontiers in Endocrinology*.

[61] Musante L., Tataruch D., Gu D. (2014). A simplified method to recover urinary vesicles for clinical applications. *Scientific Reports*.

[62] Nordin J. Z., Lee Y., Vader P. (2015). Ultrafiltration with size-exclusion liquid chromatography for high yield isolation of extracellular vesicles preserving intact biophysical and functional properties. *Nanomedicine: Nanotechnology, Biology and Medicine*.

[63] Böing A. N., van der Pol E., Grootemaat A. E., Coumans F. A. W., Sturk A., Nieuwland R. (2014). Single-step isolation of extracellular vesicles by size-exclusion chromatography. *Journal of Extracellular Vesicles*.

[64] Lozano-Ramos I., Bancu I., Oliveira-Tercero A. (2015). Size-exclusion chromatography-based enrichment of extracellular vesicles from urine samples. *Journal of Extracellular Vesicles*.

[65] Kooijmans S. A., Aleza C. G., Roffler S. R., van Solinge W. W., Vader P., Schiffelers R. M. (2016). Display of GPI-anchored anti-EGFR nanobodies on extracellular vesicles promotes tumour cell targeting. *Journal of Extracellular Vesicles*.

[66] Andreu Z., Rivas E., Sanguino-Pascual A. (2016). Comparative analysis of EV isolation procedures for miRNAs detection in serum samples. *Journal of Extracellular Vesicles*.

[67] Alvarez M. L., Khosroheidari M., Kanchi Ravi R., Distefano J. K. (2012). Comparison of protein, microRNA, and mRNA yields using different methods of urinary exosome isolation for the discovery of kidney disease biomarkers. *Kidney International*.

[68] Royo F., Diwan I., Tackett M. R. (2016). Comparative miRNA analysis of urine extracellular vesicles isolated through five different methods. *Cancers*.

[69] Wang D., Sun W. (2014). Urinary extracellular microvesicles: isolation methods and prospects for urinary proteome. *Proteomics*.

[70] Li M., Zeringer E., Barta T., Schageman J., Cheng A., Vlassov A. V. (2014). Analysis of the RNA content of the exosomes derived from blood serum and urine and its potential as biomarkers. *Philosophical Transactions of the Royal Society B: Biological Sciences*.

[71] Li P., Kaslan M., Lee S. H., Yao J., Gao Z. (2017). Progress in Exosome Isolation Techniques. *Theranostics*.

[72] Crossland R. E., Norden J., Bibby L. A., Davis J., Dickinson A. M. (2016). Evaluation of optimal extracellular vesicle small RNA isolation and qRT-PCR normalisation for serum and urine. *Journal of Immunological Methods*.

[73] Gallart-Palau X., Serra A., Sze S. K. (2016). Enrichment of extracellular vesicles from tissues of the central nervous system by PROSPR. *Molecular Neurodegeneration*.

[74] Kim J., Shin H., Kim J., Kim J., Park J. (2015). Isolation of high-purity extracellular vesicles by extracting proteins using aqueous two-phase system. *PLoS ONE*.

[75] Shin H., Han C., Labuz J. M. (2015). High-yield isolation of extracellular vesicles using aqueous two-phase system. *Scientific Reports*.

[76] Ko J., Carpenter E., Issadore D. (2016). Detection and isolation of circulating exosomes and microvesicles for cancer monitoring and diagnostics using micro-/nano-based devices. *Analyst*.

[77] Nakai W., Yoshida T., Diez D. (2016). A novel affinity-based method for the isolation of highly purified extracellular vesicles. *Scientific Reports*.

[78] Shih C.-L., Chong K.-Y., Hsu S.-C. (2016). Development of a magnetic bead-based method for the collection of circulating extracellular vesicles. *New Biotechnology*.

[79] Balaj L., Atai N. A., Chen W. (2015). Heparin affinity purification of extracellular vesicles. *Scientific Reports*.

[80] Ghosh A., Davey M., Chute I. C. (2014). Rapid isolation of extracellular vesicles from cell culture and biological fluids using a synthetic peptide with specific affinity for heat shock proteins. *PLoS ONE*.

[81] Knol J. C., de Reus I., Schelfhorst T. (2016). Peptide-mediated 'miniprep' isolation of extracellular vesicles is suitable for high-throughput proteomics. *EuPA Open Proteomics*.

[82] Royo F., Zuñiga-Garcia P., Sanchez-Mosquera P. (2016). Different EV enrichment methods suitable for clinical settings yield different subpopulations of urinary extracellular vesicles from human samples. *Journal of Extracellular Vesicles*.

[83] He M., Zeng Y. (2016). Microfluidic Exosome Analysis toward Liquid Biopsy for Cancer. *Journal of Laboratory Automation*.

[84] Dudani J., Gossett D. R., Tse H. T. K., Lamm R. J., Kulkarni R. P., Carlo D. D. (2015). Rapid inertial solution exchange for enrichment and flow cytometric detection of microvesicles. *Biomicrofluidics*.

[85] Davies R. T., Kim J., Jang S. C., Choi E.-J., Gho Y. S., Park J. (2012). Microfluidic filtration system to isolate extracellular vesicles from blood. *Lab on a Chip *.

[86] Zhao Z., Yang Y., Zeng Y., He M. (2016). A microfluidic ExoSearch chip for multiplexed exosome detection towards blood-based ovarian cancer diagnosis. *Lab on a Chip*.

[87] Caponnetto F., Manini I., Skrap M. (2016). Size-dependent cellular uptake of exosomes. *Nanomedicine: Nanotechnology, Biology and Medicine*.

[88] Puhka M., Nordberg M.-E., Valkonen S. (2017). KeepEX, a simple dilution protocol for improving extracellular vesicle yields from urine. *European Journal of Pharmaceutical Sciences*.

[89] Zeringer E., Barta T., Li M., Vlassov A. V. (2015). Strategies for isolation of exosomes. *Cold Spring Harbor Protocols*.

[90] Cheng L., Sharples R. A., Scicluna B. J., Hill A. F. (2014). Exosomes provide a protective and enriched source of miRNA for biomarker profiling compared to intracellular and cell-free blood. *Journal of Extracellular Vesicles*.

[91] Channavajjhalaa S. K., Rossatoa M., Morandini F. (2014). Optimizing the purification and analysis of miRNAs from urinary exosomes. *Clinical Chemistry and Laboratory Medicine*.

[92] Rood I. M., Deegens J. K. J., Merchant M. L. (2010). Comparison of three methods for isolation of urinary microvesicles to identify biomarkers of nephrotic syndrome. *Kidney International*.

[93] Webber J., Clayton A. (2013). How pure are your vesicles?. *Journal of Extracellular Vesicles*.

[94] Fernández-Llama P., Khositseth S., Gonzales P. A., Star R. A., Pisitkun T., Knepper M. A. (2010). Tamm-Horsfall protein and urinary exosome isolation. *Kidney International*.

[95] Wachalska M., Koppers-Lalic D., van Eijndhoven M. (2016). Protein Complexes in Urine Interfere with Extracellular Vesicle Biomarker Studies. *Journal of Circulating Biomarkers*.

[96] Chen C., Skog J., Hsu C.-H. (2010). Microfluidic isolation and transcriptome analysis of serum microvesicles. *Lab on a Chip *.

[97] Zarovni N., Corrado A., Guazzi P. (2015). Integrated isolation and quantitative analysis of exosome shuttled proteins and nucleic acids using immunocapture approaches. *Methods*.

[98] Xu R., Greening D. W., Zhu H.-J., Takahashi N., Simpson R. J. (2016). Extracellular vesicle isolation and characterization: toward clinical application. *The Journal of Clinical Investigation*.

[99] Musante L., Saraswat M., Ravidà A., Byrne B., Holthofer H. (2013). Recovery of urinary nanovesicles from ultracentrifugation supernatants. *Nephrology Dialysis Transplantation *.

[100] Pisitkun T., Johnstone R., Knepper M. A. (2006). Discovery of urinary biomarkers. *Molecular & Cellular Proteomics*.

[101] Ismail N., Wang Y., Dakhlallah D. (2013). Macrophage microvesicles induce macrophage differentiation and miR-223 transfer. *Blood*.

[102] Crescitelli R., Lässer C., Szabó T. G. (2013). Distinct RNA profiles in subpopulations of extracellular vesicles: apoptotic bodies, microvesicles and exosomes. *Journal of Extracellular Vesicles*.

[103] Campoy I., Lanau L., Altadill T. (2016). Exosome-like vesicles in uterine aspirates: A comparison of ultracentrifugation-based isolation protocols. *Journal of Translational Medicine*.

[104] Gudbergsson J. M., Johnsen K. B., Skov M. N., Duroux M. (2016). Systematic review of factors influencing extracellular vesicle yield from cell cultures. *Cytotechnology*.

[105] Chen W.-X., Liu X.-M., Lv M.-M. (2014). Exosomes from drug-resistant breast cancer cells transmit chemoresistance by a horizontal transfer of MicroRNAs. *PLoS ONE*.

[106] Hogan M. C., Johnson K. L., Zenka R. M. (2014). Subfractionation, characterization, and in-depth proteomic analysis of glomerular membrane vesicles in human urine. *Kidney International*.

[107] Graham J. M. (2001). Purification of a crude mitochondrial fraction by density-gradient centrifugation. *Current Protocols in Cell Biol*.

[108] de Araùjo M. E., Hube L. A., Stasyk T. (2008). Isolation of Endocitic Organelles by Density Gradient Centrifugation. *Methods in Molecular Biology*.

[109] Raj D. A., Fiume I., Capasso G., Pocsfalvi G. (2012). A multiplex quantitative proteomics strategy for protein biomarker studies in urinary exosomes. *Kidney International*.

[110] Chen C., Hogan M., Ward C. (2013). Purification of exosome-like vesicles from urine. *Methods in Enzymology*.

[111] Hogan M., Manganelli L., Woollard J. R. (2009). Characterization of PKD protein-positive exosome-like vesicles. *Journal of the American Society of Nephrology*.

[112] Tauro B. J., Greening D. W., Mathias R. A., Mathivanan S., Ji H., Simpson R. J. (2013). Two distinct populations of exosomes are released from LIM1863 colon carcinoma cell-derived organoids. *Molecular & Cellular Proteomics*.

[113] Cantin R., Diou J., Bélanger D., Tremblay A. M., Gilbert C. (2008). Discrimination between exosomes and HIV-1: purification of both vesicles from cell-free supernatants. *Journal of Immunological Methods*.

[114] Cantin R., Méthot S., Tremblay M. J. (2005). Plunder and stowaways: Incorporation of cellular proteins by enveloped viruses. *Journal of Virology*.

[115] Cheruvanky A., Zhou H., Pisitkun T. (2007). Rapid isolation of urinary exosomal biomarkers using a nanomembrane ultrafiltration concentrator. *American Journal of Physiology-Renal Physiology*.

[116] Merchant M. L., Powell D. W., Wilkey D. W. (2010). Microfiltration isolation of human urinary exosomes for characterization by MS. *PROTEOMICS - Clinical Applications*.

[117] Gonzales P., Pisitkun T., Knepper M. A. (2008). Urinary exosomes: Is there a future?. *Nephrology Dialysis Transplantation *.

[118] Xu R., Simpson R. J., Greening D. W. (2016). A protocol for isolation and proteomic characterization of distinct extracellular vesicle subtypes by sequential centrifugal ultrafiltration. *Methods in Molecular Biology*.

[119] Heinemann M. L., Ilmer M., Silva L. P. (2014). Benchtop isolation and characterization of functional exosomes by sequential filtration. *Journal of Chromatography A*.

[120] Muller L., Hong C.-S., Stolz D. B., Watkins S. C., Whiteside T. L. (2014). Isolation of biologically-active exosomes from human plasma. *Journal of Immunological Methods*.

[121] Baranyai T., Herczeg K., Onódi Z. (2015). Isolation of exosomes from blood plasma: Qualitative and quantitative comparison of ultracentrifugation and size exclusion chromatography methods. *PLoS ONE*.

[122] Welton J. L., Webber J. P., Botos L.-A., Jones M., Clayton A. (2015). Ready-made chromatography columns for extracellular vesicle isolation from plasma. *Journal of Extracellular Vesicles*.

[123] Yamamoto K. R., Alberts B. M., Benzinger R., Lawhorne L., Treiber G. (1970). Rapid bacteriophage sedimentation in the presence of polyethylene glycol and its application to large-scale virus purification. *Virology*.

[124] Alvarez M. L. (2014). Isolation of urinary exosomes for RNA biomarker discovery using a simple, fast, and highly scalable method. *Methods in Molecular Biology*.

[125] Gunter K. K., Gunter T. E., Jarkowski A., Rosier R. N. (1982). A method of resuspending small vesicles separated from suspension by protamine aggregation and centrifugation. *Analytical Biochemistry*.

[126] Gallart-Palau X., Serra A., Wong A. S. W. (2015). Extracellular vesicles are rapidly purified from human plasma by PRotein Organic Solvent PRecipitation (PROSPR). *Scientific Reports*.

[127] Clayton A., Court J., Navabi H. (2001). Analysis of antigen presenting cell derived exosomes, based on immuno-magnetic isolation and flow cytometry. *Journal of Immunological Methods*.

[128] Oksvold M. P., Neurauter A., Pedersen K. W. (2015). Magnetic bead-based isolation of exosomes. *Methods in Molecular Biology*.

[129] Peterson M. F., Otoc N., Sethi J. K., Gupta A., Antes T. J. (2015). Integrated systems for exosome investigation. *Methods*.

[130] Ueda K., Ishikawa N., Tatsuguchi A., Saichi N., Fujii R., Nakagawa H. (2014). Antibody-coupled monolithic silica microtips for highthroughput molecular profiling of circulating exosomes. *Scientific Reports*.

[131] Chen C., Lin B.-R., Hsu M.-Y., Cheng C.-M. (2015). Paper-based devices for isolation and characterization of extracellular vesicles. *Journal of Visualized Experiments*.

[132] Enderle D., Spiel A., Coticchia C. M. (2015). Characterization of RNA from exosomes and other extracellular vesicles isolated by a novel spin column-based method. *PLoS ONE*.

[133] Ingato D., Lee J. U., Sim S. J., Kwon Y. J. (2016). Good things come in small packages: Overcoming challenges to harness extracellular vesicles for therapeutic delivery. *Journal of Controlled Release*.

[134] Théry C., Zitvogel L., Amigorena S. (2002). Exosomes: composition, biogenesis and function. *Nature Reviews Immunology*.

[135] Miyanishi M., Tada K., Koike M., Uchiyama Y., Kitamura T., Nagata S. (2007). Identification of Tim4 as a phosphatidylserine receptor. *Nature*.

[136] Sharon N., Lis H. (2004). History of lectins: from hemagglutinins to biological recognition molecules. *Glycobiology*.

[137] Echevarria J., Royo F., Pazos R., Salazar L., Falcon-Perez J. M., Reichardt N.-C. (2014). Microarray-based identification of lectins for the purification of human urinary extracellular vesicles directly from urine samples. *ChemBioChem*.

[138] Kosanović M., Janković M. (2014). Isolation of urinary extracellular vesicles from Tamm-Horsfall protein–depleted urine and their application in the development of a lectin-exosome-binding assay. *BioTechniques*.

[139] Evander M., Gidlöf O., Olde B., Erlinge D., Laurell T. (2015). Non-contact acoustic capture of microparticles from small plasma volumes. *Lab on a Chip *.

[140] Wang Z., Wu H.-J., Fine D. (2013). Ciliated micropillars for the microfluidic-based isolation of nanoscale lipid vesicles. *Lab on a Chip *.

[141] Liga A., Vliegenthart A. D. B., Oosthuyzen W., Dear J. W., Kersaudy-Kerhoas M. (2015). Exosome isolation: A microfluidic road-map. *Lab on a Chip *.

[142] Iorember F. M., Vehaskari V. M. (2014). Uromodulin: Old friend with new roles in health and disease. *Pediatric Nephrology*.

[143] Youhanna S., Weber J., Beaujean V., Glaudemans B., Sobek J., Devuyst O. (2014). Determination of uromodulin in human urine: Influence of storage and processing. *Nephrology Dialysis Transplantation *.

[144] Serafini-Cessi F., Malagolini N., Cavallone D. (2003). Tamm-Horsfall glycoprotein: Biology and clinical relevance. *American Journal of Kidney Diseases*.

[145] Kobayashi K., Fukuoka S. (2001). Conditions for solubilization of Tamm-Horsfall protein/ uromodulin in human urine and establishment of a sensitive and accurate enzyme-linked immunosorbent assay (ELISA) method. *Archives of Biochemistry and Biophysics*.

[146] Akiyama A., Stein P. C., Houshiar A., Parsons C. L. (2000). Urothelial cytoprotective activity of Tamm-Horsfall protein isolated from the urine of healthy subjects and patients with interstitial cystitis. *International Journal of Urology*.

[147] Argade S., Chen T., Shaw T. (2015). An evaluation of Tamm–Horsfall protein glycans in kidney stone formers using novel techniques. *Urolithiasis*.

[148] Musante L., Saraswat M., Duriez E. (2012). Biochemical and physical characterisation of urinary nanovesicles following CHAPS treatment. *PLoS ONE*.

[149] Bokhove M., Nishimura K., Brunati M. (2016). A structured interdomain linker directs self-polymerization of human uromodulin. *Proceedings of the National Acadamy of Sciences of the United States of America*.

[150] Gámez-Valero A., Lozano-Ramos S. I., Bancu I., Lauzurica-Valdemoros R., Borràs F. E. (2015). Urinary extracellular vesicles as source of biomarkers in kidney diseases. *Frontiers in Immunology*.

[151] Pisitkun T., Shen R.-F., Knepper M. A. (2004). Identification and proteomic profiling of exosomes in human urine. *Proceedings of the National Acadamy of Sciences of the United States of America*.

[152] Chen C. Y., Hogan M. C., Ward C. J. (2013). Purification of exosome-like vesicles from urine. *Methods in Enzymology*.

